# Conjugative plasmid-encoded toxin–antitoxin system PrpT/PrpA directly controls plasmid copy number

**DOI:** 10.1073/pnas.2011577118

**Published:** 2021-01-22

**Authors:** Songwei Ni, Baiyuan Li, Kaihao Tang, Jianyun Yao, Thomas K. Wood, Pengxia Wang, Xiaoxue Wang

**Affiliations:** ^a^Key Laboratory of Tropical Marine Bio-resources and Ecology, Guangdong Key Laboratory of Marine Materia Medica, Innovation Academy of South China Sea Ecology and Environmental Engineering, South China Sea Institute of Oceanology, Chinese Academy of Sciences, Nansha District, 511458 Guangzhou, China;; ^b^Southern Marine Science and Engineering Guangdong Laboratory (Guangzhou), Nansha District, 511458 Guangzhou, China;; ^c^Key Laboratory of Comprehensive Utilization of Advantage Plants Resources in Hunan South, College of Chemistry and Bioengineering, Hunan University of Science and Engineering, 425199 Yongzhou, Hunan, China;; ^d^Department of Chemical Engineering, The Pennsylvania State University, University Park, PA 16802-4400;; ^e^University of Chinese Academy of Sciences, 100049 Beijing, China

**Keywords:** toxin–antitoxin, plasmid replication, plasmid copy number, ParE, origin of replication

## Abstract

Toxin–antitoxin (TA) loci were initially identified on conjugative plasmids, and one function of plasmid-encoded TA systems is to stabilize plasmids or increase plasmid competition via postsegregational killing. Here, we discovered that the type II TA system, *Pseudoalteromonas rubra* plasmid toxin–antitoxin PrpT/PrpA, on a low-copy-number conjugative plasmid, directly controls plasmid replication. Toxin PrpT resembles ParE of plasmid RK2 while antitoxin PrpA (PF03693) shares no similarity with previously characterized antitoxins. Surprisingly, deleting this *prpA*-*prpT* operon from the plasmid does not result in plasmid segregational loss, but greatly increases plasmid copy number. Mechanistically, the antitoxin PrpA functions as a negative regulator of plasmid replication, by binding to the iterons in the plasmid origin that inhibits the binding of the replication initiator to the iterons. We also demonstrated that PrpA is produced at a higher level than PrpT to prevent the plasmid from overreplicating, while partial or complete degradation of labile PrpA derepresses plasmid replication. Importantly, the PrpT/PrpA TA system is conserved and is widespread on many conjugative plasmids. Altogether, we discovered a function of a plasmid-encoded TA system that provides new insights into the physiological significance of TA systems.

## Significance

Since conjugative plasmids are usually large and may carry genes encoding functions that are detrimental to the bacterial host, minimizing plasmid copy number is critical for reducing the host burden. Toxin–antitoxin (TA) systems are one of the conserved modules on conjugative plasmids. Here, we demonstrate the functional significance of a large group of antitoxins on conjugative plasmids: the antitoxin acts as an unexpected player in the negative control of plasmid replication. For the plasmid-encoded PrpT/PrpA TA system, the antitoxin can control toxin production by binding to PrpT and by reducing plasmid copy number. This work shows that the antitoxin can directly regulate plasmid replication, expanding our understanding of the physiological role of TA systems.

Conjugative plasmids are extrachromosomal genetic elements that carry genetic determinants for adaptive traits enabling host bacteria to colonize diverse environments. They are maintained in bacterial communities through both vertical inheritance and horizontal transfer (1, 2). These plasmids are relatively large (>30 kb) and are usually kept at low copy numbers to minimize the metabolic load on bacterial hosts (3). To ensure the success of vertical and horizontal transmission, conjugative plasmids tend to include core regions required for replication, partition and other stability functions, and conjugative transfer (4, 5).

For conjugative plasmids, replication and partition are of utmost importance for their maintenance in bacterial populations (5). Initiation of plasmid DNA replication requires a specific plasmid-encoded Rep initiator protein and a specific plasmid origin of replication with which Rep interacts (6). After plasmid replication, the partition system directs plasmid copies to daughter cells (5). Postsegregational killing (PSK) is an additional strategy to ensure plasmid stability by killing those rare cells that lose the plasmid due to replication or segregation errors (5).

Toxin–antitoxin (TA) systems were originally discovered on conjugative plasmids in the 1980s and later they were found to be ubiquitous among conjugative plasmids (7). TA systems were first proposed to play a role in plasmid stability through PSK by eliminating plasmid-free cells to ensure plasmid vertical inheritance (8–10). However, the stability hypothesis alone cannot explain the success of PSK-encoding plasmids (11) or the maintenance of multiple TA systems on plasmids (12). The more recent competition hypothesis proposes that TA systems have been selected on plasmids through horizontal plasmid propagation rather than due to vertical propagation (11). The competition hypothesis is strongly supported by the fact that a plasmid encoding the ParE/ParD TA system excluded an isogenic plasmid devoid of this TA module under conditions of horizontal gene transfer (11), and competition between plasmids led to a higher accumulation of TA systems on plasmids relative to chromosomes when transposon-encoded TA systems were added (13). Nevertheless, since their discovery, relatively little work has been conducted to explore the functions of plasmid-based TA systems beyond plasmid stability and competition. Compared to chromosomally encoded TA systems, the function of plasmid-encoded TA systems in various cellular processes has been largely overlooked.

TA systems are classified into seven types, of which type II TA systems are the most abundant in bacterial genomes and plasmids (14). Toxins cause a wide range of cellular effects including inhibition of translation and replication, as well as disruption of cell membrane integrity (15). A typical type II TA system comprises two genes located in an operon that encodes a stable toxin and a labile antitoxin. In type II TA systems, antitoxins generally are composed of two independent domains, an N-terminal DNA-binding domain and a C-terminal toxin-binding domain. The type II antitoxin forms tight complexes with the respective toxin to neutralize its activity, and TA system expression is tightly autoregulated by the antitoxin alone or by the TA complex (7). The regulatory function depends on the DNA-binding domain that binds to the specific sequence of the operon promoter region to repress transcription of the TA system (7). In addition to the autoregulation of the TA operon, increasing evidence shows that antitoxins can regulate other gene loci. We previously demonstrated that type II antitoxin MqsA in *Escherichia coli* binds to other gene loci to regulate the stress response (through binding to the promoter of *rpoS*) and biofilm formation (through binding to the promoter *csgD*) (16, 17) and that the type II antitoxin HigA in *Pseudomonas aeruginosa* binds to the promoter of the *mvfR* to regulate virulence (18). Further study of the antitoxins should provide additional insights into the physiological roles of TA systems.

ParE-type toxins are highly abundant in both plasmids and bacterial chromosomes, and the RK2-encoded ParE/ParD TA system is known to help maintain plasmid RK2 (19). The coupled antitoxins of ParE toxins are usually annotated as ParD based on “guilt by association” even though they share low or no similarity with ParD in RK2 (PFAM: PF09386). By bioinformatic analyses, here we found that PF03693 family proteins associated with the ParE toxin are much more abundant than those of the PF09386 family. Recently, we identified a ParE/PF03693 TA in the conjugative plasmid pMBL6842 in the marine bacterium *Pseudoalteromonas rubra* (20). By studying the ParE/PF03693 pair (renamed here as PrpT/PrpA) in pMBL6842, we found that this plasmid is stably maintained at 2 copies per cell, has a ParAB partition system, and has a well-controlled replication system. Deleting the *prpAT* TA operon of pMBL6842 did not result in segregational plasmid loss, but surprisingly, the deletion caused plasmid overreplication and led to a very high copy number. Moreover, we found that the antitoxin PrpA directly binds to the iterons in the plasmid origin, which could hinder the binding of the Rep protein to the iterons. Thus, unlike the previously studied ParE/PF09386 TA system of RK2, the *P. rubra* ParE/PF03693 TA pair of pMBL6842 has a novel function in regulating plasmid replication. In addition, ParE/PF03693 pairs are often found in large conjugative plasmids of pathogenic and environmental bacteria. Hence, our results expand our understanding of the physiological role of plasmid-encoded TA systems.

## Results

### Antitoxins Associated with Toxin ParE Belong to Multiple PFAM Families.

**Fig. 1.**
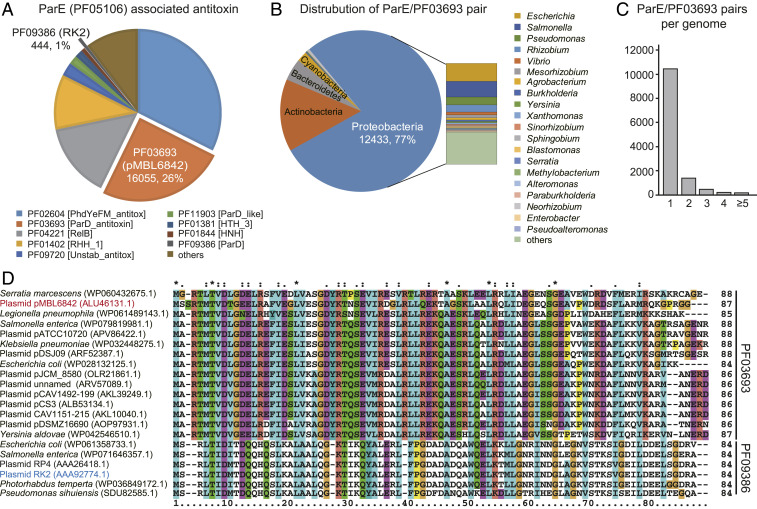
ParE/PF03693 pairs are abundant on conjugative plasmids. (*A*) ParE toxins are associated with multiple antitoxin PFAM families. Each PFAM is represented by a specific color. (*B*) Distribution of ParE/PF03693 pairs in representative phyla and genera. (*C*) The average number of ParE/PF03693 pairs per genome. (*D*) Multiple sequence alignment constructed by ClustalW to compare the amino acid sequence identity of ParE associated antitoxins from PF03693 and PF09386 in the conjugative plasmids.

To gain insights into antitoxin function, we analyzed the sequences of ParE-associated antitoxins. We retrieved 104,865 ParE toxins from the IMG/M database, and identified 62,457 ParE-associated antitoxins accordingly (Dataset S1). In general, the ParE toxins were more conserved than their cognate antitoxins, since all ParE toxins belong to one PFAM family (PF05016). In contrast, the cognate antitoxins of these ParE toxins were associated with multiple PFAM families (Fig. 1*A*). Surprisingly, the PF09386 family, which was initially named “ParD” and includes the well-characterized ParD antitoxin of plasmid RK2, includes only ∼1% of all ParE-associated antitoxins. Instead, the largest antitoxin grouping is PF02604 (∼33%) which is closely related to the Phd/YeFM superfamily antitoxin. The second-largest grouping is the PF03693 family (∼26%), but proteins from this family (also named ParD) share no similarity with ParD of the PF09386 family. Furthermore, ParE/PF03693 pairs are widely distributed in Proteobacteria, and they are also found in Actinobacteria, Bacteroidetes, and Cyanobacteria (Fig. 1*B* and Dataset S2). Some strains also contain multiple ParE/PF03693 pairs in their genomes (Fig. 1*C*). By further searching the available sequenced plasmids, we found that ParE/PF03693 pairs are often found in conjugative plasmids of pathogenic and environmental bacteria, including *Salmonella enterica, Enterobacter kobei,* and *P. rubra* (Fig. 1*D* and *SI Appendix*, Table S1). However, the function of ParE/PF03693 on conjugative plasmids remains unexplored.

### PrpT Is a Potent Toxin and PrpT/PrpA Constitute a Type II TA Pair.

**Fig. 2.**
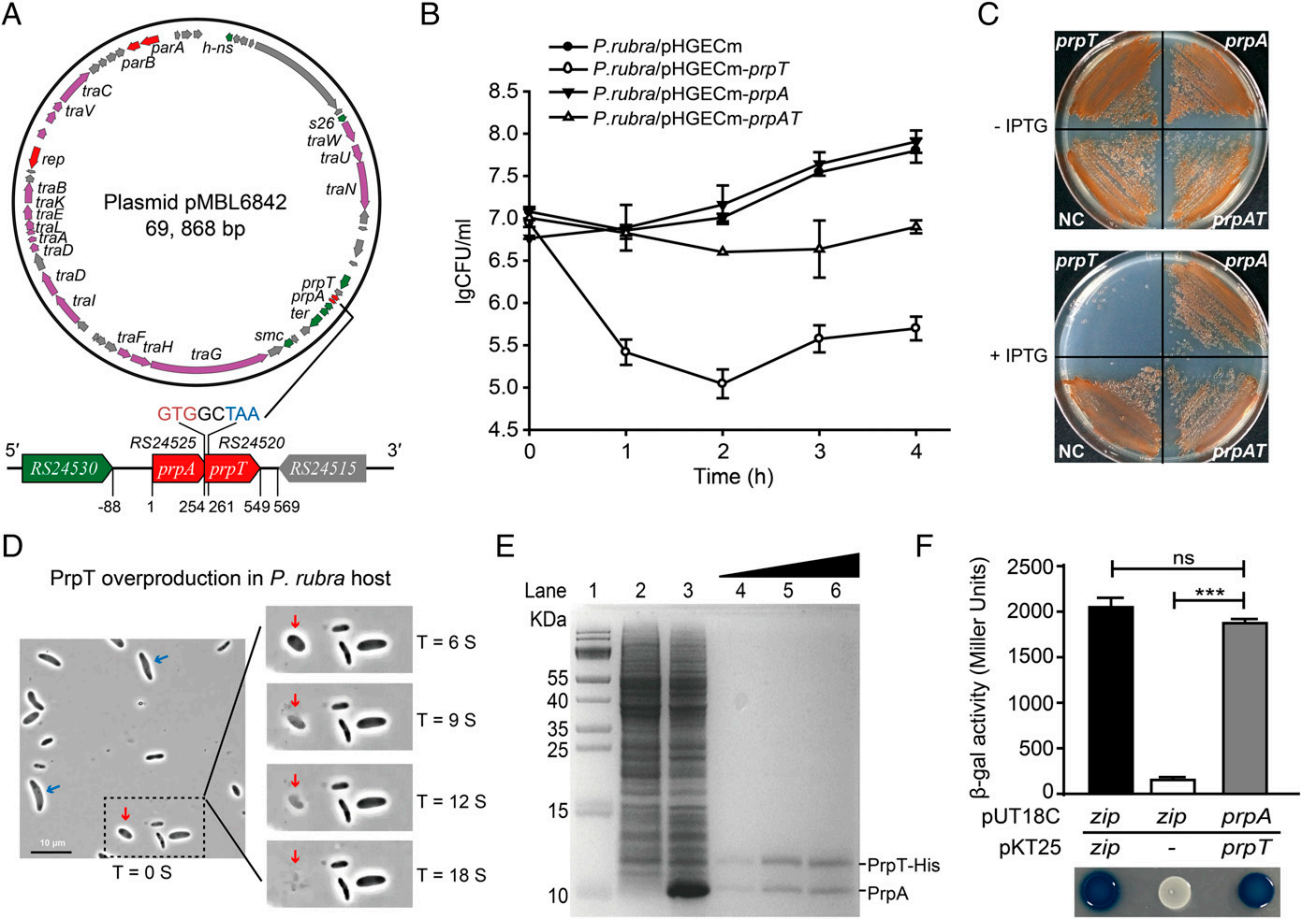
PrpT and PrpA constitute a TA pair. (*A*) Circular map of pMBL6842 and the position of the *prpA*-*prpT* operon. (*B* and *C*) Viability of cells overexpressing *prpT*, *prpA,* and *prpA*-*prpT* in *P. rubra*. IPTG, isopropyl β-ᴅ-thiogalactoside. (*D*) Morphologies of cells overexpressing *prpT* with 0.5 mM IPTG for 2 h over time (see Movie S1). The ghost and lysed cells are marked with blue and red arrows. (*E*) PrpT and PrpA form a complex in vitro. His-tagged PrpT and untagged PrpA were coproduced via pET28b-*prpA*-*prpT*-His (lane 3) and copurified with increasing concentration of imidazole (lanes 4–6). Lane 1: size marker; lane 2: NC (no IPTG). (*F*) The BACTH assay showed that PrpT interacts with PrpA. The data are from three independent cultures. SDs are shown, and statistical significance (NS, no significant; *P < 0.05; **P < 0.01; ***P < 0.0001) is indicated with asterisks in Figs. 2–4 and 6. Images shown in Figs. 2–6 are representative images.

To conduct a functional study, the ParE/PF03693 pair from the conjugative plasmid pMBL6842 in *P. rubra* was characterized as a representative ParE/PF03693 TA pair. Plasmid pMBL6842 is 69.9 kb and carries the replication initiator RepB, plasmid partition proteins ParA/ParB, and 18 conjugation-transfer–related proteins (Fig. 2*A*). In pMBL6842, two neighboring genes, AT705_RS24520 and AT705_RS24525, were identified as a putative TA pair belonging to ParE/PF03693 (*SI Appendix*, Figs. S1 and S2). To avoid confusion with ParE/ParD, we propose to name this TA pair as PrpT/PrpA (*P. rubra* plasmid toxin–antitoxin). *prpT* encodes a protein of 98 aa, and the upstream gene *prpA* encodes a protein of 86 aa (Fig. 2*A*). PrpT has 49% sequence similarity with ParE_RK2_, while PrpA has no sequence similarity with ParD_RK2_ (PF09386) (Fig. 1). PrpA shares amino acid 51–80% sequence similarity with the unstudied PF03693 proteins in other conjugative plasmids from diverse bacterial strains (*SI Appendix*, Table S1).

To test whether PrpT and PrpA constitute a TA pair, the toxicity of these genes was first tested by individually expressing *prpT* and *prpA* in the original host. Overexpression of PrpT resulted in severe growth inhibition and cell death (Fig. 2 *B* and *C* and *SI Appendix*, Fig. S3*A*). We also examined the morphology of the *P. rubra* cell overexpressing *prpT*. PrpT overproduction resulted in “ghost” cell morphology (Fig. 2*D*, marked with blue arrows), which indicates a dead or dying cell with a dense cell pole and a transparent center (21). Notably, the ghost cell can further undergo cell lysis and the whole cell is ruptured within seconds (refer to Movie S1 for the process of cell lysis; the lysed cell is marked with a red arrow in Fig. 2*D*). Furthermore, we also tested the toxicity of PrpT and PrpA in *E. coli*. Similar to *P. rubra*, PrpT overproduction in *E. coli* also resulted in growth inhibition and cell death (*SI Appendix*, Fig. S3*B*). Unlike in *P. rubra*, overproduction of PrpT caused filamentous growth in *E. coli* (*SI Appendix*, Fig. S3*C*). In contrast, PrpA production did not affect cell growth in either host, and it could completely neutralize the toxic effect of PrpT in both bacterial hosts (Fig. 2 *B* and *C* and *SI Appendix*, Fig. S3 *A* and *B*).

The coding regions of *prpA* and *prpT* overlap by eight bases (Fig. 2*A* and *SI Appendix*, Fig. S3*D*), and the two genes were cotranscribed (*SI Appendix*, Fig. S3*E*). We performed a pull-down assay to determine whether PrpT and PrpA form a complex in vitro. PrpT with a C-terminal hexa-histidine tag (His-tag) was produced together with the untagged PrpA. Sodium dodecyl sulfate polyacrylamide gel electrophoresis (Tricine-SDS-PAGE) revealed an ∼10-kDa protein that copurified with His-tagged PrpT at a ratio of ∼1:1 (Fig. 2*E*). Mass spectrometry analysis verified that the copurified protein was PrpA. Next, a Cya-based bacterial two-hybrid (BACTH) assay confirmed that PrpT directly interacts with PrpA (Fig. 2*F*). Taken together, we found that PrpT and PrpA constitute a type II TA pair in which PrpT is a potent toxin and PrpA is the cognate antitoxin.

### PrpA Autoregulates the *prpA*-*prpT* Operon.

**Fig. 3.**
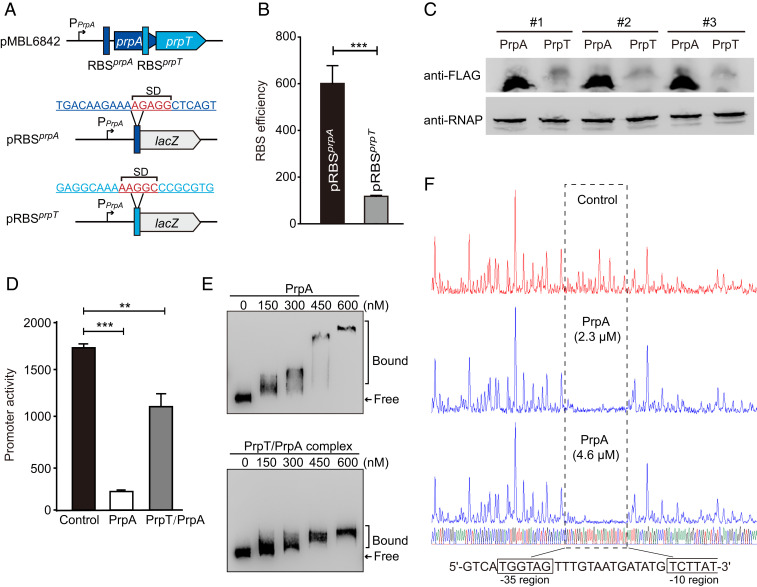
PrpA and PrpT/PrpA bind to the *prpA*-*prpT* operon. (*A*) Schematic of the *prpA*-*prpT* operon. The Shine–Dalgano sequences of *prpA* and *prpT* are highlighted in red. (*B*) Comparison of the RBS activities is shown using the two *lacZ* reporter plasmids, pRBS^*prpA*^ or pRBS^*prpT*^ in *A*. (*C*) Western blot showing that the production of PrpA exceeded PrpT (*n* = 3); the results were obtained by using FLAG-tagged PrpA (10.5 kDa) or PrpT (12.3 kDa). RNAP was used as a control. (*D*) The promoter activity was measured by overexpressing PrpA or PrpT/PrpA using pRBS^*prpA*^. (*E*) EMSA results showed that PrpA and PrpA/PrpT complex bound and shifted the promoter in a dose-dependent manner. (*F*) The binding site of PrpA is analyzed by the DNase I footprinting assay using two different concentrations of PrpA. The 30-bp binding site of PrpA covers the −35 and partial −10 regions of the promoter.

Although *prpA* and *prpT* form an operon and are cotranscribed, the ribosomal binding sites (RBSs) of the two genes are different (Fig. 3*A* and *SI Appendix*, Fig. S3*D*), suggesting that the toxin and antitoxin might be translated at different levels. To compare the production of PrpA and PrpT driven by their native RBS, two mosaic plasmids, pRBS^*prpA*^ and pRBS^*prpT*^, were constructed to measure the RBS efficiency of each mRNA in *E. coli*. The activity was 601 ± 80 MU for the RBS^*prpA*^ and 117 ± 4 MU for RBS^*prpT*^, indicating that the translation of PrpA was more efficient than PrpT (Fig. 3*B*). Additionally, we also constructed FLAG-tagged PrpA (tag at the N terminus) and PrpT (tag at the C terminus) produced from their own RBS to measure the protein production in *E. coli* by Western blot analysis, and the results showed that PrpA was produced at a higher level than PrpT (Fig. 3*C*).

In most type II TA systems, the antitoxin and/or the TA complex bind DNA and autoregulate the transcription of the TA operon (7). PrpA contains a ribbon–helix–helix (RHH) domain (*SI Appendix*, Fig. S1*A*) which can confer DNA binding (22). Using the pRBS^*prpA*^ plasmid with the native promoter as the reporter, we found that the promoter activity was decreased from 1,730 ± 40 MU to 198 ± 17 MU after producing PrpA. Moreover, coexpressing *prpA* and *prpT* also significantly decreased the promoter activity (Fig. 3*D*). Next, we performed an electrophoretic mobility shift assay (EMSA) using purified PrpA to determine whether PrpA directly binds to the promoter in vitro. As shown in Fig. 3*E*, PrpA bound and shifted the promoter in a dose-dependent manner. The PrpT/PrpA complex also shifted the promoter (Fig. 3*E*), which agrees with the results of the in vivo β-galactosidase assay. Next, we performed a DNase I footprinting assay to identify the binding site of PrpA. The results show that a single 30-bp region is protected from DNase I digestion by PrpA, and the binding site of PrpA is located within the putative −35 and −10 regions of the promoter (Fig. 3*F*). Altogether, the in vivo and in vitro assays show that antitoxin and the TA complex can bind to the promoter region and repress the TA operon.

### PrpA Regulates Plasmid Copy Number.

**Fig. 4.**
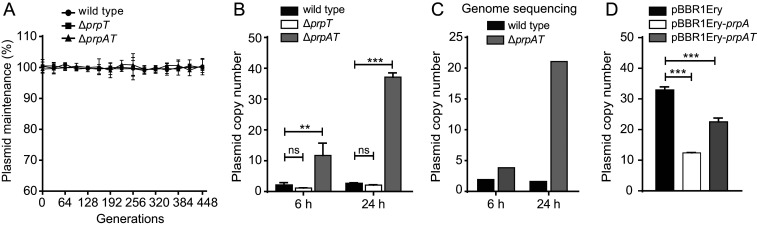
PrpA controls plasmid copy number. (*A*) PrpT/PrpA TA system does not control the segregational stability of plasmid pMBL6842. The wild-type and deletion mutants were cultured in 2216E medium without antibiotic for 448 generations. Quantification of plasmid copy number after cultivation for 6 h (in the exponential phase) and 24 h (in the late stationary phase) from the starting point OD_600_ ∼0.01, by qPCR (*n* = 4) (*B*) and by PCR-free whole-genome sequencing (*C*). (*D*) Quantification of the plasmid copy number when expressing *prpA* and *prpAT* under their native promoter in the Δ*prpAT* strain after 24 h by qPCR (*n* = 4).

To test whether the PrpT/PrpA TA system resembles ParE/ParD of plasmid RK2 in terms of contributing to plasmid stability, we first deleted *prpT* and *prpA*-*prpT* from pMBL6842 in *P. rubra* (*SI Appendix*, Fig. S4*A*). These deletions did not alter *P. rubra* growth significantly (*SI Appendix*, Fig. S4*B*). When *prpT* or the TA operon was deleted, no loss of plasmid pMBL6842 was detected for 448 generations (cells reinoculated every 16 generations) (Fig. 4*A*). Surprisingly, the plasmid copy number was greatly increased when the TA operon was deleted based on qPCR quantification. The plasmid copy number in the *prpAT* deletion mutant strain was 12 ± 4 copies per cell after 6 h (in the exponential phase), while it remained at 2.1 ± 0.8 copies per cell in the wild-type strain. After 24 h (in the late stationary phase), the copy number in the *prpAT* deletion mutant reached 37 ± 1 copies per cell, while it remained low (1.9 ± 0.7 copies per cell) in the wild-type strain (Fig. 4*B*).

Furthermore, the plasmid copy number was determined using a PCR-free, whole-genome sequencing approach which has been used to quantify the copy number of a large conjugative plasmid in *Yersinia* sp (23). The plasmid copy numbers in the *prpAT* deletion mutant were 4 and 21 copies per cell after cultivation of 6 and 24 h, while it remained at ∼2 copies per cell in the wild-type strain. By contrast, deletion of *prpT* alone did not affect the plasmid copy number, suggesting that deletion of the antitoxin *prpA* was responsible for the increased plasmid copy number in the *prpAT* deletion mutant (Fig. 4*C*). Notably, comparing the empty vector, expressing *prpA* in the *prpAT* deletion mutant using the native promoter significantly reduced the plasmid copy number from 33 ± 1 to 12 ± 1 copies per cell, and coexpressing of *prpT* and *prpA* significantly reduced the plasmid copy number to 23 ± 1 copies per cell (Fig. 4*D*). Collectively, these results demonstrate that PrpT/PrpA reduces the plasmid copy number in *P. rubra* and that PrpA is directly responsible for this effect.

### PrpA Binds to the Iterons in the Plasmid Origin to Inhibit Replication Initiation.

Plasmid pMBL6842 is a stringently regulated plasmid, and our results show it is stably maintained at 1–2 copies per cell in growth conditions without selection pressure, suggesting that the plasmid should contain well-controlled replication machinery. In most cases, low-copy-number large plasmids, such as P1, F, RK2, and R6K replicate by the theta mode (6). Iterons are repeated initiator binding sites in the plasmid *ori* and are crucial for replication initiation (24). We found pMBL6842 encodes a putative initiator protein RepB. As expected, deleting *repB* led to a complete loss of pMBL6842 (*SI Appendix*, Fig. S5*A*). To explore whether the TA system regulates the transcription of *repB*, *lacZ* was fused to the *repB* promoter to make the reporter plasmid pHGR01-P_*repB*_. The promoter activity assay showed that neither PrpT/PrpA nor PrpA regulated the promoter of *repB* (*SI Appendix*, Fig. S5*B*). Next, to determine the replication origin, a 2.4-kb fragment (976-bp upstream region and the coding of *repB* gene) was inserted into the *E. coli* cloning vector pHGM01 which does not replicate in *P. rubra*, generating pRepB1. We found that pRepB1 can replicate in *P. rubra*, suggesting that 2.4-kb fragment contains functional replication machinery. Four truncated fragments (pRepB2-B5) were then tested, and only pRepB2 which contains a 397-bp sequence upstream of *repB* replicated in *P. rubra* (*SI Appendix*, Fig. S6*A*). Hence, the 397-bp sequence contains a functional *ori*.

**Fig. 5.**
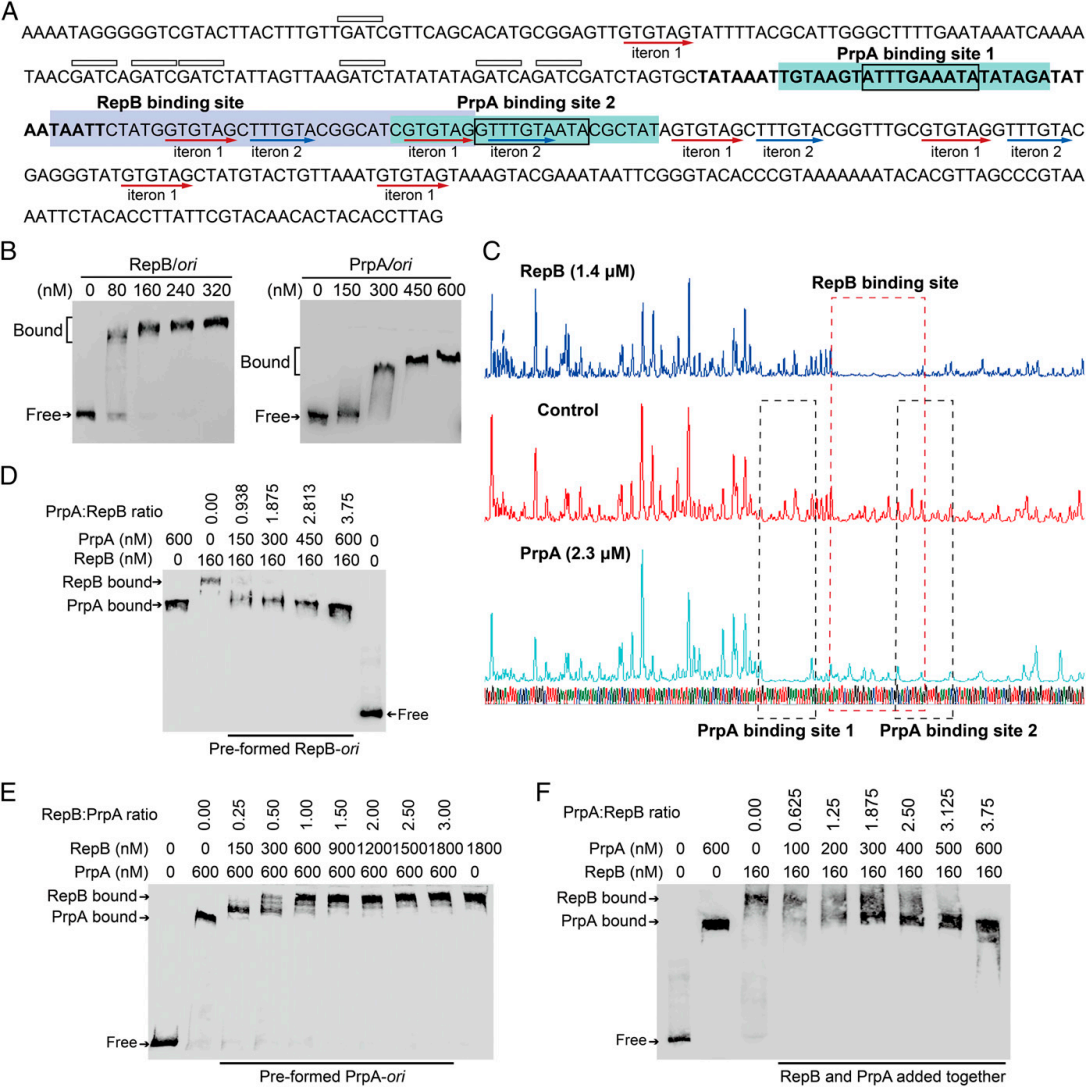
PrpA competes with RepB for binding to the pMBL6842 *ori*. (*A*) Nucleotide sequence of the pMBL6842 *ori* region. The AT-rich region is marked in bold letters. The 5′-GATC sites are indicated by boxes above the sequence. The iterons are underlined using red arrows. The binding sites of RepB and PrpA are highlighted with blue and green, respectively. The conserved binding motif of PrpA in site 1 and 2 is indicated by a box in the sequences. (*B*) EMSA results showing that RepB and antitoxin PrpA bind and shift the pMBL6842 *ori*. (*C*) DNase I footprinting assays used to determine the binding sites of RepB and PrpA. EMSA results showing that PrpA and RepB compete for binding to *ori* when the two proteins are added sequentially (*D* and *E*) or added at the same time (*F*).

pMBL6842 *ori* contains a 44-bp element with 89% AT content followed by an array of iterons. In particular, the array of iterons contain seven copies of iteron 1 (5′-GTGTAG-3′) and four copies of iteron 2 (5′-TTTGTA-3′). A signature 5′-GATC-3′ sequence that is usually found in the initiation of DNA replication (6) is also abundant upstream of the iterons (Fig. 5*A*). EMSA and DNase I footprinting assays were performed using purified RepB and the origin sequence to search for the binding sites of RepB. We found that RepB can bind to the origin in a dose-dependent manner and that the binding site is a 36-bp sequence containing two copies of iteron 1 and one copy of iteron 2, indicating these iterons are critical for replication initiation (Fig. 5 *B* and *C*). To determine whether PrpA binds to the plasmid *ori*, we performed EMSA assays using purified PrpA and the PrpT/PrpA complex. The results show that PrpA binds and shifts the *ori* (Fig. 5*B*), but PrpA does not bind control DNA (*SI Appendix*, Fig. S6*B*). Moreover, DNase I footprinting revealed that the binding sites of PrpA cover iteron 1 and iteron 2 (Fig. 5*C*), suggesting PrpA may compete with RepB for binding to *ori*. To further test this, competition assays were performed, and the EMSA assay results show that PrpA outcompeted the binding of RepB to *ori* when added at a ratio of PrpA/RepB ≥ 1 (Fig. 5*D*), and could not effectively displace RepB bound to *ori* at a ratio of PrpA/RepB < 1 (*SI Appendix*, Fig. S6*C*). Similarly, the addition of RepB can outcompete the binding of PrpA to *ori* when added at a ratio of RepB/PrpA ≥ 1 (Fig. 5*E*). Moreover, when the two proteins were added at the same time, they competed for the binding to *ori* (Fig. 5*F*). These results indicate there is competitive binding of PrpA and RepB to *ori*. A conserved motif [5′-TTTG(T/A)AAT-3′] is located in the PrpA binding site of the *prpAT* promoter and in the binding sites of the plasmid origin (*SI Appendix*, Fig. S6*D*). As expected, the binding of PrpA to a mutated *ori* with the iterons disrupted was greatly reduced (*SI Appendix*, Fig. S6*E*). Additionally, unlike the binding of PrpT/PrpA complex to its own promoter, the PrpT/PrpA complex does not bind the plasmid *ori* (*SI Appendix*, Fig. S6*F*). Altogether, our results demonstrate that PrpA and RepB compete for binding to the iterons of *ori*.

### PrpA Has a Modular Structure and Is Degraded at Both Termini.

**Fig. 6.**
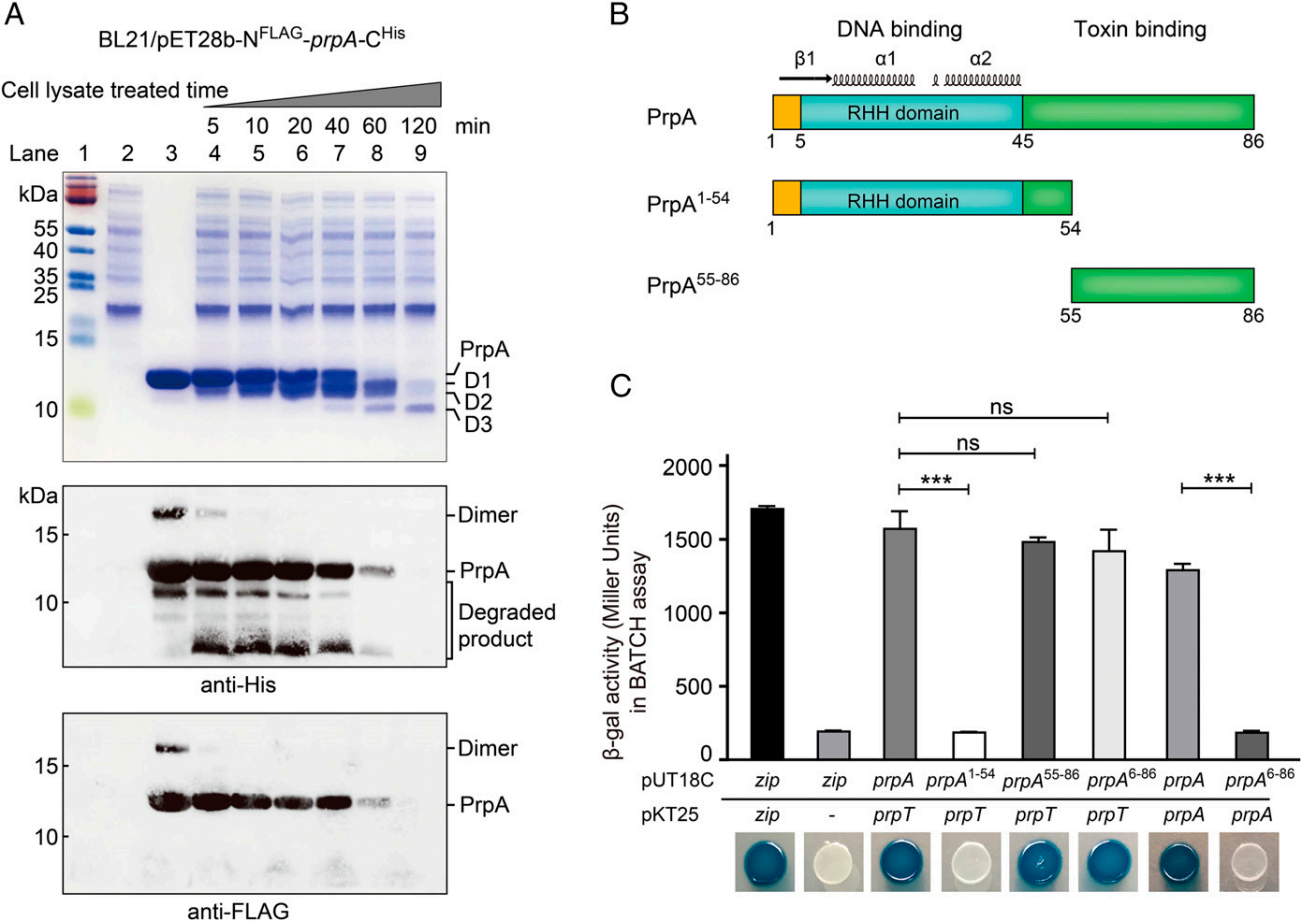
PrpA is labile and degradation occurs at both termini. (*A*) The stability of N^FLAG^-PrpA-C^His^ was determined by Tricine-SDS-PAGE (*Upper*), Western blot using anti-His antibody (*Middle*), and anti-FLAG antibody (*Lower*) after being treated with *P. rubra* cell lysates. Lane 1, size marker; lane 2, cell lysate; lane 3, purified N^FLAG^-PrpA-C^His^, lanes 4–9, N^FLAG^-PrpA-C^His^ treated with *P. rubra* lysates over time. (*B*) Schematic of the modular organization of PrpA. (*C*) A BACTH assay was performed to assess interactions between PrpA proteins of varying lengths and PrpT.

Since PrpA is responsible for controlling pMBL6842 replication in *P. rubra*, the stability of PrpA was determined. To monitor the degradation of PrpA at the two termini, we fused an FLAG-tag to the N terminus and a His-tag to the C terminus. Stationary-phase *P. rubra* cells were collected and lysed, and the whole cell lysate was used for degrading PrpA. N^FLAG^-PrpA-C^His^ was successfully purified using nickel resin, and PrpA was cleaved gradually from 5 to 120 min (Fig. 6*A*). Notably, more than half of the full-length PrpA was degraded after 40 min, and it was almost completely degraded after 60 min. Furthermore, Western blotting analysis using an antibody against the PrpA His-tag to detect the C terminus indicated that the degraded products should retain the C termini although these small fragments were invisible on SDS-PAGE. Degradation at the N terminus was detected by using an anti-FLAG tag antibody which indicated that the PrpA N terminus was degraded quickly. Thus, full-length PrpA was completely degraded after 60 min, and degradation of PrpA occurs primarily at the N terminus. Corroborating these Western blotting results, in-gel trypsin digestion followed by matrix-assisted laser desorption ionization time-of-flight mass spectrometry (MALDI-TOF MS) analysis of the cleaved bands of the SDS-PAGE (Fig. 6*A*, lane 8) revealed that the cleaved bands D1, D2, and D3 were all from PrpA. Additionally, these cleaved PrpA contain the N terminus (except the first 4 aa, which could not be determined due to enzyme digestion) (*SI Appendix*, Fig. S7*A*). The smallest cleaved product D3 was ∼5.8 kDa and the end of the C terminus of D3 was estimated to locate between 53 and 57 aa of PrpA (*SI Appendix*, Fig. S7 *B* and *C*).

Structure predictions indicate that PrpA should have a modular organization, in which the N terminus adopts an RHH DNA binding motif of the CopG family while the C terminus is relatively unstructured (Fig. 6*B* and *SI Appendix*, Fig. S8 *A* and *B*). To determine whether the N terminus is sufficient for the DNA binding, we constructed a truncated PrpA (PrpA^1–54^) with its C terminus removed. The EMSA assays show that PrpA^1–54^ can bind as efficiently as full-length PrpA to the plasmid *ori* (*SI Appendix*, Fig. S8*C*). In addition, PrpA^1–54^ can also bind and shift the *prpAT* promoter in the absence of the C terminus as shown by both EMSA and the *lacZ* prompter assays (*SI Appendix*, Fig. S8 *C* and *D*). Next, we determined whether the C terminus is sufficient for toxin binding by constructing a truncated PrpA (PrpA^55–86^) with the N terminus removed. Using a bacterial two-hybrid assay, we found that PrpA^55–86^ can bind as efficiently as the full-length PrpA to PrpT (Fig. 6*C*). In addition, we found that full-length PrpA forms dimers; however, PrpA^6-86^ does not interact with full-length PrpA but it still interacts with toxin PrpT (Fig. 6*C*), suggesting that the first 5 aa in the N terminus of PrpA are crucial for its dimerization. Thus, the degradation of PrpA at the N terminus will prevent DNA binding. Collectively, these results demonstrate PrpA has a modular organization with an N-terminus DNA binding domain that is important for regulating plasmid replication.

## Discussion

In this study, using a representative of the type II ParE/PF03693 TA pair from a conjugative plasmid, we discovered that the antitoxin of the PrpT/PrpA TA pair acts as a negative regulator of plasmid replication. Conjugative plasmids may carry genes encoding functions that are detrimental to the bacterial host; thus it is important to keep them at low copy numbers to minimize host burden. For example, PrpT overproduction is highly toxic to the bacterial host; thus, it appears that antitoxin PrpA controls PrpT production in multiple ways, by directly binding to PrpT, by autoregulating the TA operon, and by directly reducing plasmid copy number.

**Fig. 7.**
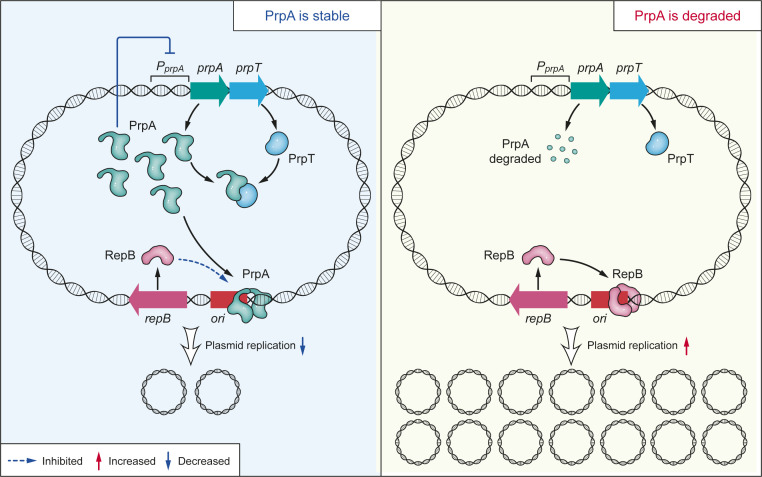
Proposed mechanism underlying how PrpT/PrpA controls plasmid replication. When PrpA is stable, it binds to the iterons in the *ori,* interfering with the binding of RepB to the *ori,* thus preventing overreplication of the plasmid. During stress, PrpA is degraded, thus derepressing the inhibition of the binding of RepB to *ori*.

Strict control of plasmid replication is achieved by tight coordination of the Rep protein and the negative control systems. The negative control systems usually involve regulating Rep production directly by antisense RNAs and transcriptional repressors (6, 25–28). The interaction between the Rep protein and iterons is essential for the initiation of plasmid replication in the theta mode, as in plasmids F and P1, and is also important for the prevention of plasmid overreplication (24, 25, 29, 30). The pMBL6842 origin shares a high similarity of organization with the P1 origin and contains multiple iterons. Indeed, we found that pMBL6842 RepB binds to the iterons in the *ori*. More importantly, we found that PrpA binds to the iterons and prevents plasmids from overreplicating by directly interfering with the interaction between RepB and the iterons. A schematic of how the PrpT/PrpA TA system controls the plasmid copy number in *P. rubra* is shown in Fig. 7. Plasmid-encoded TA systems are known to stabilize plasmids after replication and partitioning, or to increase plasmid competition during horizontal gene transfer (4, 5, 11). The pMBL6842 plasmid carries partition module ParA/ParB to minimize segregational loss after replication. Here we found that PrpT/PrpA does not contribute to the segregational loss of pMBL6842. We report that a plasmid-encoded TA system directly regulates plasmid replication, expanding our understanding of the physiological role of plasmid-encoded TA systems.

Antitoxin PrpA can effectively neutralize the toxic effect of PrpT, and it is less stable than PrpT. The molecular basis of PSK relies on the toxic effect of the toxin as well as the differential stabilities of the toxin and antitoxin (31). In plasmid-free cells, the unstable antitoxin PrpA decays, leading to the activation of PrpT and cell stasis or killing. Thus, this TA system may also help stabilize plasmids if replication or segregation errors occur. For chromosomes and plasmids, studies have revealed there is cooperation between stability/segregation modules and replication modules to ensure DNA maintenance. Indeed, chromosomal partitioning ParA influences replication of the *Bacillus subtilis* chromosome and *Vibrio cholerae* chromosome I, and ParB encoded by the *V. cholerae* chromosome II also influences chromosome II replication (32–34).

Here, we found that PrpA is produced at a higher level than PrpT which can explain how coexpressing *prpA* and *prpT* using the native promoter could reduce plasmid copy number in the TA deletion mutant. Measurements of the synthesis rates of 12 type II TA systems of *E. coli* revealed that the antitoxin is synthesized at a much higher rate than the toxin (35). Similarly, our previous study also found that the production of antitoxin HigA is much higher than toxin HigB in *P. aeruginosa,* and free HigA proteins bind to the *mvfR* promoter to regulate virulence (18). Type II antitoxins usually contain an RHH or helix–turn–helix domain which confers DNA binding at the N terminus (36). Antitoxin PrpA has a modular organization, and the N terminus of PrpA adopts a CopG-like regulatory domain (*SI Appendix*, Fig. S8*A*). CopG is one of the smallest transcriptional repressors with an RHH domain, similar to the regulatory repressors of Mnt, Arc, and MetJ (37). In fact, CopG was first named since it regulates plasmid copy number. In the streptococcal plasmid pMV158, *copG* and *repB* are cotranscribed, and CopG binds to the promoter of *copG*-*repB* to regulate plasmid replication through RepB (38). Indeed, in plasmid pMBL6842, *prpA* and *prpT* form an operon and are cotranscribed, and PrpA autoregulates the *prpA*-*prpT* operon. Different from CopG in pMV158, PrpA has an additional C terminus which confers protein binding to the respective toxin. Nevertheless, our truncation studies showed the N terminus (1–54 aa) of PrpA is sufficient for the binding of PrpA to its own promoter and to the plasmid *ori*. From an evolutionary standpoint, our results suggest that type II antitoxins may have evolved from small transcriptional repressors, and the role of PrpA in modulating copy number appears to be one of many costrategies by which it acts as an antitoxin.

Plasmid copy number is dynamic in the bacterial life cycle and modulating plasmid copy number is the key for conjugative plasmids to live in harmony with their host bacteria (26). It has been reported that nutrient limitation at late stationary phase may lead to a rested chromosomal DNA replication but plasmid production could be still running (39). Here we found that the TA-system-bearing plasmid pMBL6842 is maintained at 1–2 copies per cell during different growth phases, while plasmid copy number increases in the Δ*prpAT* strain with culture age. These results suggest that this TA system is involved in synchronizing plasmid and chromosome replication through the interaction of the antitoxin with the plasmid *ori*. Our analysis found that ParE/PF03693 TA pairs are also found in antibiotic resistance and virulence plasmids (*SI Appendix*, Table S1), which implies this TA pair may also be important for copy number control of these plasmids. In support of this intriguing idea, the up-regulation of the copy number of a ParE/PF03693 TA containing a virulence plasmid encoding for a type III secretion system (T3SS) is essential for *Yersinia pseudotuberculosis* to establish infections (copy number increases from ∼1 copy per cell to 6 copies per cell), while high T3SS expression is deleterious for cell growth (23). Therefore, additional investigations are warranted to explore whether this TA pair regulates the replication of virulence plasmids during infection. It would also be important to explore whether plasmid-encoded antitoxins are engaged in the regulation of virulence and antibiotic resistance genes in addition to acting as the antidote for the toxin.

## Materials and Methods

### Strains, Plasmids, Segregation Stability Assay, and Plasmid Origin Identification.

The deletion mutants were constructed following the protocols described previously (40). The strains, plasmids, and primers used in this study are listed in *SI Appendix*, Tables S2 and S3. The details of plasmid construction, the segregation stability assay, and plasmid origin identification are described in *SI Appendix*. The ParE-associated antitoxins (Datasets S1 and S2) were analyzed using the function profile tool in the IMG/M system (41).

### Protein Purification, PrpA Degradation, EMSA, BACTH, Western Blotting, and DNase I Footprinting Assay.

Ni-NAT resin, anti-FLAG, anti-His, and anti-RNA polymerase (RNAP) antibodies were used for protein purification and the Western blot. The purified PrpA were treated with *P. rubra* cell lysates collected at the early stationary (optical density OD_600_ ∼3.0) over time. The EMSA, BACTH assay, and DNase I footprinting assay are described in *SI Appendix*.

### Quantification of Plasmid Copy Number.

Plasmid copy number was quantified by qPCR and also by a PCR-free whole-genome sequencing approach previously described (23). For details, see *SI Appendix*.

## Supplementary Material

Supplementary File

Supplementary File

Supplementary File

Supplementary File

## Data Availability

All study data are included in the article and/or supporting information.
