# Supplementary material for: Conjugative plasmid-encoded toxin–antitoxin system PrpT/PrpA directly controls plasmid copy number
Source: Proc Natl Acad Sci U S A. 2021 Jan 22;118(4):e2011577118. doi: 10.1073/pnas.2011577118 (PMC7848731; doi:10.1073/pnas.2011577118)
Supplement: Supplementary File [file pnas.2011577118.sapp.pdf]

SUPPLEMENTARY FILE

Conjugative Plasmid-Encoded Toxin-Antitoxin System PrpT/PrpA Directly Controls Plasmid Copy Number

Songwei Ni[1, 2#], Baiyuan Li[3#], Kaihao Tang[1,2], Jianyun Yao[1,2], Thomas K. Wood[4], Pengxia Wang[1,2,5*], Xiaoxue Wang[1,2,5*]

*To whom correspondence should be addressed: Xiaoxue Wang, E-mail: xxwang@scsio.ac.cn or Pengxia Wang, wangpengxia@scsio.ac.cn

This SI PDF file includes:

- **S1 Materials and Methods**
- **Figures S1 to S7**
- **Tables S1 to S3**
- **SI References**

Legends for the other three supplementary materials for this manuscript

- **Movie S1.** Morphologies of cells overexpressing *prpT* via pHGECm-*prpT* with 0.5 mM IPTG for 2 h were recorded by phase contrast microscopy over time.
- **Dataset S1.** The PFAM families of antitoxins associated with ParE (PF05016) toxins. 66,524 ParE-associated antitoxins, which were 70-120 aa length were analyzed.
- **Dataset S2.** Detailed information for PF03693 family antitoxins associated with ParE. The conjugative plasmids containing ParE/PF03693 pairs are highlighted in yellow and can be screened by "Plasmid.Bacteria" in Domain.

S1 Materials and Methods

Bacterial strains, plasmids, and growth conditions. The bacterial strains, plasmids and primers used in this study are listed in **Table S2** and **Table S3**. *P. rubra* was grown in Marine Broth 2216E medium (Difco) at 25°C, and the *E. coli* strains were grown in Luria broth (LB) at 37°C unless specified otherwise. DAP (2,6-diamino-pimelic acid) (50 μg/mL) was added when culturing the *E. coli* WM3064 strain. Ampicillin (100 μg/ml) was used for maintaining pUT18C-based and pMD19-T-based plasmids, kanamycin (50 μg/mL) was used for maintaining pKT25-based, pHGR01-based and pET28b-based plasmids in *E. coli*, chloramphenicol (30 μg/ml) was used for maintaining pCA24N-based plasmids, chloramphenicol (15 μg/ml) was used for maintaining pHGECm-based plasmids, Erythrocin (25 μg/ml) was used for maintaining pBBR1Ery-based plasmids and gentamicin (30 μg/ml) was used for maintaining pHGM01-based plasmids. IPTG (isopropyl-*β*-D-thiogalactopyranoside) (1 mM for protein purification, 0.5 mM for reporter activity assay) was used as an inducer.

Analysis of the distribution of ParE/PF03693 pairs. Using the function profile tool in the IMG/M system (http://genome.jgi.doe.gov/, Version 5.1 Sep. 2019) (1), a total of 62,457 ParE-associated antitoxins with PFAM filtered by 70-120 aa length were retrieved. The cognate antitoxins of these ParE toxins were associated with multiple PFAM families, including 20,803 PF02604 [PhdYeFM_antitox], 16,055 PF03693 [ParD_antitoxin], 9,249 PF04221 [RelB], 6,821 PF01402 [RHH_1], and 444 PF09386 [ParD] (**Dataset S1**). The distribution of the PF03693/ParE pairs in representative phyla and genera were further analyzed (**Dataset S2**), and the plasmids with "Plasmid: Bacteria" Domain was recovered from the IMG/M database (**Table S1**).

Construction of deletion mutants in *P. rubra*. The deletion mutants were constructed as described previously in *Pseudoalteromonas* (2). Briefly, the upstream and downstream regions of *prpT* and *prpA-prpT* operon were amplified by PCR from *P. rubra* genomic DNA with primers listed in **Table S3**. The PCR fragments were fused and ligated into pHGM01 (3) using the ClonExpress II One Step Cloning Kit (Vazyme, Nanjing, China), producing suicide plasmids pHGM01-*prpT* and pHGM01-*prpAT*. Similarly, the upstream and downstream regions of *rep* were amplified from SCSIO 6842 genomic DNA with primer pairs RepB-up-F/-R and RepB-down-F/-R and ligated into pHGM01, producing suicide plasmid pHGM01-*repB*. To monitor the plasmid pMBL6842, the Cm resistance gene cassette was amplified using primers Cm-F/Cm-R from plasmid pCA24N. The upstream and downstream regions of the targeted loci for Cm integration were amplified from SCSIO 6842 genomic DNA with primers Cm-up-F/-R, Cm-down-F/-R. The three PCR fragments were fused and ligated into pHGM01, generating suicide plasmid pHGM01-*cm*. Then, the suicide plasmids pHGM01-*prpT*, pHGM01-*prpAT*, pHGM01-*repB* and pHGM01-*cm* were respectively transferred from *E. coli* WM3064 into SCSIO 6842 by conjugation experiments using a previously described method (2). The deletion mutants were obtained via homologous recombination by sucrose resistance selection method. Finally, the mutants were confirmed by PCR and DNA sequencing.

Construction of plasmids. The pHGECm vector was used to express target genes in *P. rubra* and pCA24N was used to express target genes in *E. coli*. The pBBR1Ery clone vector was used to complement *prpA* or the *prpAT* operon with its own promoter in *P. rubra* Δ*prpAT* strain. The pHGR01 vector was used to construct the *lacZ* reporter plasmids. The pET28b vector was used to purify the targeted proteins or quantify the production of targeted proteins by Western blot in *E. coli.* The pKT25 and pUT18C vectors were used to test the interaction of two proteins in BACTH assays. In detail, coding region of *prpT*, *prpA* or the *prpAT* operon was amplified from *P. rubra* with primer pairs pHGECm-PrpT-F/-R, pHGECm-PrpA-F/-R or pHGECm-PrpA-F/pHGECm-PrpT-R respectively, PCR product was digested with *EcoRI/XhoI* and inserted into the corresponding sites of pHGECm using the T4 DNA Ligase, generating inducible pHGECm-*prpT*, pHGECm-*prpA* and pHGECm-*prpAT*. Coding region of *prpT*, *prpA* or the *prpAT* operon was amplified from SCSIO 6842 with primer pairs pCA24N-PrpT-F/-R, pCA24N-PrpA-F/-R, pCA24N-PrpA-F/pCA24N-PrpT-R respectively, PCR product was ligated into the *StuI* digested pCA24N vector using the ClonExpress II One Step Cloning Kit (Vazyme Biotech Co.,Ltd), generating inducible pCA24N-*prpT*, pCA24N-*prpA* and pCA24N-*prpAT*. Coding region of *prpA* or the *prpAT* operon with their own promoter was amplified from SCSIO 6842 with primer pairs pBBR1Ery-PrpA-F/-R or pBBR1Ery-PrpA-F/pBBR1Ery-PrpAT-R respectively, PCR product was

digested with *EcoRI/XhoI* and inserted into the corresponding sites of pBBR1Ery using the T4 DNA Ligase, generating complementary plasmids pBBR1Ery-*prpA* and pBBR1Ery-*prpAT*. To generate $pRBS^{prpA}$ and $pRBS^{prpT}$ reporter plasmids, promoter region of *prpAT* was amplified from SCSIO 6842 with primer pairs $pRBS^{prpA}$-F/-R, PCR product was used as template and amplified with primer pairs $pRBS^{prpA}$-F/ $pRBS^{prpT}$-R, two PCR products were digested with *Eco*RI/*Hin*dIII and inserted into the corresponding sites of pHGR01. To generating plasmid expressing Flag-Tagged PrpA or Flag-Tagged PrpT driven by T7*lac* promoter their native RBS, *prpAT* operon containing RBS^{prpA} (do not containing P_{prpA})was amplified and fused with Flag-tag (N-terminal of PrpA or C-terminal of PrpT) using primer pairs listed in **Table S3**, final PCR product was inserted into *XbaI* and *NcoI* digested pET28b vector, generating pET28b-RBS^{prpA}-N^{FLAG}-PrpAT and pET28b-RBS^{prpA}-PrpAT-C^{FLAG}. Other plasmids were constructed with similar method, all constructions were finally confirmed by PCR and DNA sequencing.

Conjugation and transformation. pHGECm-based and pBBR1Ery-based expression vectors were transferred into *P. rubra* from *E. coli* WM3064 by conjugation. pCA24N-based expression vectors and pHGR01-based promoter-reporter plasmids were electroporated into *E. coli* K-12 BW25113, pET28b-based expression vectors were electroporated into *E. coli* BL21 (DE3). The pKT25- and pUT18C-based vectors were electroporated into *E. coli* BTH101.

Reporter activity assay. Specific *β*-galactosidase activity of strains harboring the *prpAT* or *repB* promoter-reporter plasmids was determined by monitoring the absorbance at 420 nm using the Miller assay (4). To determine the promoter activity of *prpAT* and *repB* under overexpression of *prpA*, *prpA-prpT*, $prpA^{1\text{-}54}$ and $prpA^{55\text{-}86}$, pCA24N-*prpA*, pCA24N-*prpAT,* pCA24N-$prpA^{1\text{-}54}$ or pCA24N-$prpA^{55\text{-}86}$ was transformed into the *E. coli* host carrying the reporter. Overnight cultures were diluted 1:100 in LB with Kan and Cm, and induced with 0.5 mM IPTG at an OD_{600} of 0.5. After induction for 6 h, cells were collected to determine the *β*-galactosidase activity. To compare the translation efficiency driven by RBS^{prpA} and RBS^{prpT}, strains carrying the *lacZ* reporter plasmid $pRBS^{prpA}$ or $pRBS^{prpT}$ were diluted 1:100 in LB with Kan after overnight culture and collected at an OD_{600} of 1.5, and then, *β*-galactosidase activity was determined.

Identification pMBL6842 origin (*ori*). A 2.4 kb PCR product was amplified with primer pairs pRepB1-F/-R and was cloned into the *E. coli* cloning vector pHGM01 which does not replicate in *P. rubra* to make pRepB1. Plasmid pRepB2 was generated by amplifying two PCR products with primer pairs pRepB1-F/pRepB2-mid-R and pRepB2-mid-F/pRepB1-R and cloning into pHGM01. The other three truncated fragments were amplified with primer pairs pRepB3-F/pRepB1-R, pRepB4-F/pRepB1-R, and pRepB1-F/pRepB5-R, and cloned into pHGM01, producing plasmids pRepB3, pRepB4 and pRepB5, respectively. These plasmids were then transferred into plasmid-free strain *P. rubra* ΔpMBL6842 from *E. coli* WM3064 by conjugation, and were selected by plating on 2216E agar plates containing gentamicin.

Determination generation time. Generation time (G_t) of cells in culture is defined as the time required for the cell number to double, accordingly, G_t of *P. rubra* was measured following previously described protocols (5). In detail, three independent colonies of strain SCSIO 6842 were grown overnight in 2216E medium at 25°C. th e overnight cultures were diluted to an OD_{600} of 0.1 in fresh 2216E medium, cells for 0 h, 1 h, 2 h and 3 h were collected and serially diluted in 10-fold, 10 µl was dropped on 2216E plates and incubated at 25°C for 24 h, the colony-forming units (CFUs) were recorded. The generation time was calculated by the formula: $G_t = [\log(N) - \log(N_0)] \div K$, where N would equal 2 and N_0 would equal 1 (or N = 4, $N_0 = 2$, or any other values representing a doubling in number), K representing the slope of the line during CFU increase along with culture time. The results showed that the generation time of *P. rubra* is 45.8 ± 3.6 min per generation in 2216E medium at 25°C.

Segregational stability assay. Strains of *P. rubra* containing plasmid pMBL6842*::cm* or its derivatives pMBL6842*::cm*Δ*prpA*, pMBL6842*::cm*Δ*prpAT* were grown in 2216E medium to determine the contribution of PrpT/PrpA TA system to plasmid stability. An 2216E preculture with Cm was used to inoculate 3 ml 2216E without antibiotic. Every 12 h of growth (~16 generations), bacterial suspensions were diluted 1000-fold in 3 ml fresh 2216E medium. The cultures were serially diluted in 10-fold dilution steps from days 1 to 14, and 10 µl was dropped on 2216E plates with or without Cm. The CFU assay was conducted every day (~32 generations) for 14 days, and the number of CFUs was determined.

Each experiment was performed in triplicate with three independent cultures.

Quantification of plasmid copy number. Plasmid copy number of pMBL6842 in *P. rubra* WT and TA deletion mutants were quantified by quantitative PCR (qPCR) and also by a whole-genome sequencing approach previously described (6). Overnight cultures of *P. rubra* WT and TA deletion mutants were diluted into OD_{600}~0.01 and cultured in 2216E medium for 24 h. Cells in exponential (~6 h) and late stationary (~24 h) phases were collected to determine the plasmid copy number of pMBL6842. Total DNA was extracted using a TIANamp Bacteria DNA kit (Tiangen, China) following manufacturer's instructions. The number of chromosomes was quantified based on the chromosomally-encoded single-copy gene *gyrB*, and the number of pMBL6842 plasmids was quantified based on the plasmid-encoded single-copy gene *repB*. Furthermore, we also employed a PCR-free, whole-genome sequencing approach, where the ratio of coverage depth between plasmid DNA and chromosomal DNA reflects the copy number of pMBL6842 in the wild-type and Δ*prpAT* strains. In the complementation study, pBBR1Ery-based plasmids expressing PrpA or the PrpT/PrpA complex under their native promoter were transferred into *P. rubra* Δ*prpAT* from *E. coli* WM3064, after 24 h immediate culture in 2216E with Ery when correct conjugants were obtained, plasmid copy number of pMBL6842 was quantified by the similar qPCR method.

Protein purification and mass spectrometry analysis. Protein PrpA, PrpT/PrpA complex, $PrpA^{1-54}$, N^{FLAG}-*prpA*-C^{His} and RepB were purified from *E. coli* BL21 (DE3) containing the targeted plasmids pET28b-*prpA*-His, pET28b-*prpAT*-His, pET28b-*prpA*$^{1-54}$-His, pET28b-N^{FLAG}-*prpA*-C^{His} and pET28b-*repB*-His. Briefly, the overnight cultures were diluted to an OD_{600} of 0.05 in fresh LB with IPTG (1 mM) was added at OD_{600} of 0.5, and one liter of the cells were collected after induction for 5 h. The subsequent steps of protein extraction from the collected pellet were performed as previously described (7). The purified proteins were desalted by passage through Sephadex G-25 pre-packed PD-10 columns, and protein concentrations were quantified using a Bi Yuntian BCA assay kit (Haimen, China). Tricine-SDS-PAGE was performed as previously described (8). For subsequent mass spectrometry analysis, protein bands were excised and analyzed by BGI Tech Solutions Co., Ltd. (Shenzhen, China). Peptide mass fingerprints were analyzed using the MASCOT search program version 2.3.01 (9) and the previously annotated *P. rubra* genome (GenBank accession numbers CP013611 to CP013613) (10) as the reference database.

Electrophoretic mobility shift assay (EMSA). EMSA was carried out as described previously (11) to evaluate the binding effects of purified protein on corresponding biotin-labelled DNA probe using the LightShift^{TM} EMSA Optimization and Control Kit (Thermo, USA). The *prpAT* promoter and the *ori* were PCR amplified from the SCSIO 6842 genomic DNA using primer pairs P_{prpAT}-probe-F/-R and ori-probe-F/-R, mutant *ori* was amplified and fused using primer pairs listed in **Table S3**, the PCR product was gel-purified and cloned into pMD19-T, generating pMD19-T-P_{prpAT}, pMD19-T-*ori* and pMD19-T-*oriGC*. Biotin-labeled M13F and ordinary M13R were used to amplify the mature probe. Empty pMD19-T was used to amplify the control probe. For the binding reactions, DNA probe (0.1 pmol) was added to different amounts of purified protein in 20 μl system containing 2 μl 10×Binding buffer, 1 μl 50% Glycerol, 1 μl 1% NP40, 1 μl 1M KCl, 1 μl 100 mM $MgCl_2$ and 0.25 μl 1μg/μl Poly (dI:dC). The binding reactions were incubated at 25 °C for 2 h and loaded onto a 6% DNA retardation gel at 110 V in 0.5 ×TBE [45 mM Tris (pH 8.3), 45 mM boric acid and 1 mM EDTA] and subjected to electrophoresis at 4 °C for 60 min. DNA probe was transferred to a nylon membranes at 400 mA for 30 min and subsequently UV-crosslinked at 302 nm for 20 min. Stabilized streptavidin-HRP (50 μl) was added and the mixture was incubated at room temperature for 30 min in Blocking Buffer. After washing with 1×Wash Buffer for three times, the chemiluminescent signal was detected using Luminol/Enhancer Solution and Nucleic Acid Detection Blocking Buffer according to the manufacturer's protocol. To test the competition of PrpA and RepB for their binding effect to *ori*, one protein was first added at a fixed concentration (RepB-160 nM, PrpA-600 nM) into the reaction system and incubation for 20 minutes at 25 °C, the other protein was then added at a gradually increased concentration (PrpA-from 0 to 600 nM, RepB-from 0 to 1800 nM) to compete with the first one for the binding to *ori*, after incubation at 25 °C for another 40 min, reaction products were loaded onto 6% DNA retardation gel and followed the procedure as described ablove (**Fig. 5DE**). To assess whether adding order of PrpA and RepB has a critical effect on competition binding assay, we also performed a parallel experiment in which PrpA and

RepB were added simultaneously (**Fig. 5F**).

Western blot analysis. To quantify the production of PrpA or PrpT, overnight cultures of *E. coli* strains BL21/pET28b-RBS^{prpA}-N^{FLAG}-PrpAT and BL21/ pET28b-RBS^{prpA}-PrpAT- C^{FLAG} were diluted 100-fold in fresh LB with Kan and IPTG. Cells in stationary (~12 h) were collected. To monitor the degradation of PrpA at the N and C termini, strain BL21/pET28b-N^{FLAG}-PrpA-C^{His} was collected and used to purify proteins. Equal purified proteins were incubated with stationary-phase *P. rubra* cell lysates for varying amounts of time. Then, total protein was loaded, and tricine-SDS-PAGE was performed. The proteins were transferred to a PVDF membrane, Western blot was performed as described previously, and the antibodies were provided by the same supplier (12). Briefly, primary antibodies raised against a His-tag or Flag-tag and horseradish-peroxidase-conjugated goat anti-mouse secondary antibodies were used in the assays. The expression of anti-RNA polymerase beta antibody (RNAP) was used as a reference in all samples, and the horseradish peroxidase-conjugated goat anti-rabbit antibody was used as a secondary antibody.

Bacterial two-hybrid (BACTH) assay. The pKT25 and pUT18C plasmids carrying different target-cloned inserts were co-transformed into *E. coli* BTH101 (*cya−99*) competent cells. Co-transformed cells were plated on LB plates supplemented with ampicillin, kanamycin, IPTG and X-gal at final concentrations of 100 µg/ml, 50 µg/ml, 0.5 mM or 40 µg/ml, respectively. The corrected cells were cultivated at 30°C for 12-24 h. The pKT25-*zip* and pUT18C-*zip* plasmids were used as positive controls (Euromedex), and the pKT25 (without insert) and pUT18C-*zip* (fused with a leucine zipper protein) plasmids were used as negative controls (13).

DNase I footprinting assay. FAM-labeled probes were amplified with FAM-labeled M13F and ordinary M13R from pMD19-T-P$_{prpAT}$ and pMD19-T-*ori*. For each reaction, 5 pmol FAM-labeled probes was added to purified protein (50 times more than used in EMSA reaction) in a 50 µl system containing 5 µl 10 × Binding Buffer, 2.5 µl 50% Glycerol, 2.5 µl 1% NP40, 2.5 µl 1M KCl, 2.5 µl 100 mM $MgCl_2$ and 0.625 µl 1µg/µl Poly (dI:dC). All binding reactions were incubated at 25 °C for 2 h, 1/8 U DNase I was then added to digest the DNA probe for about 4 min, 5 µl 200 mM EDTA was used to stop the digestion and DNA probe was purified using a QIAquick® Nucleotide Removal Kit (QIAGEN, Germany). Products were subjected to STR analysis to identify protected sites by corresponding protein.

Protein degradation assay. *P. rubra* cell lysates were prepared using early stationary *P. rubra* cells (OD_{600} ~ 3.0). Cell pellets were collected by centrifugation at 10,000 g for 6 min at 4°C and washed twice with ice-cold lysis buffer (50 mM potassium phosphate buffer and 300 mM NaCl). The FastPrep-24™ homogenizer was used to homogenize cells at 20 s intervals five times, and cell debris was further removed by centrifugation at 10,000 g for 10 min at 4°C. Purified PrpA was incubated with *P. rubra* cell lysates at 25°C for varying amounts of time. Tricine-SDS-PAGE gels were first used to evaluate the degradation profiles of PrpA, and then Western blot analysis was used to determine which PrpA terminus was cleaved.

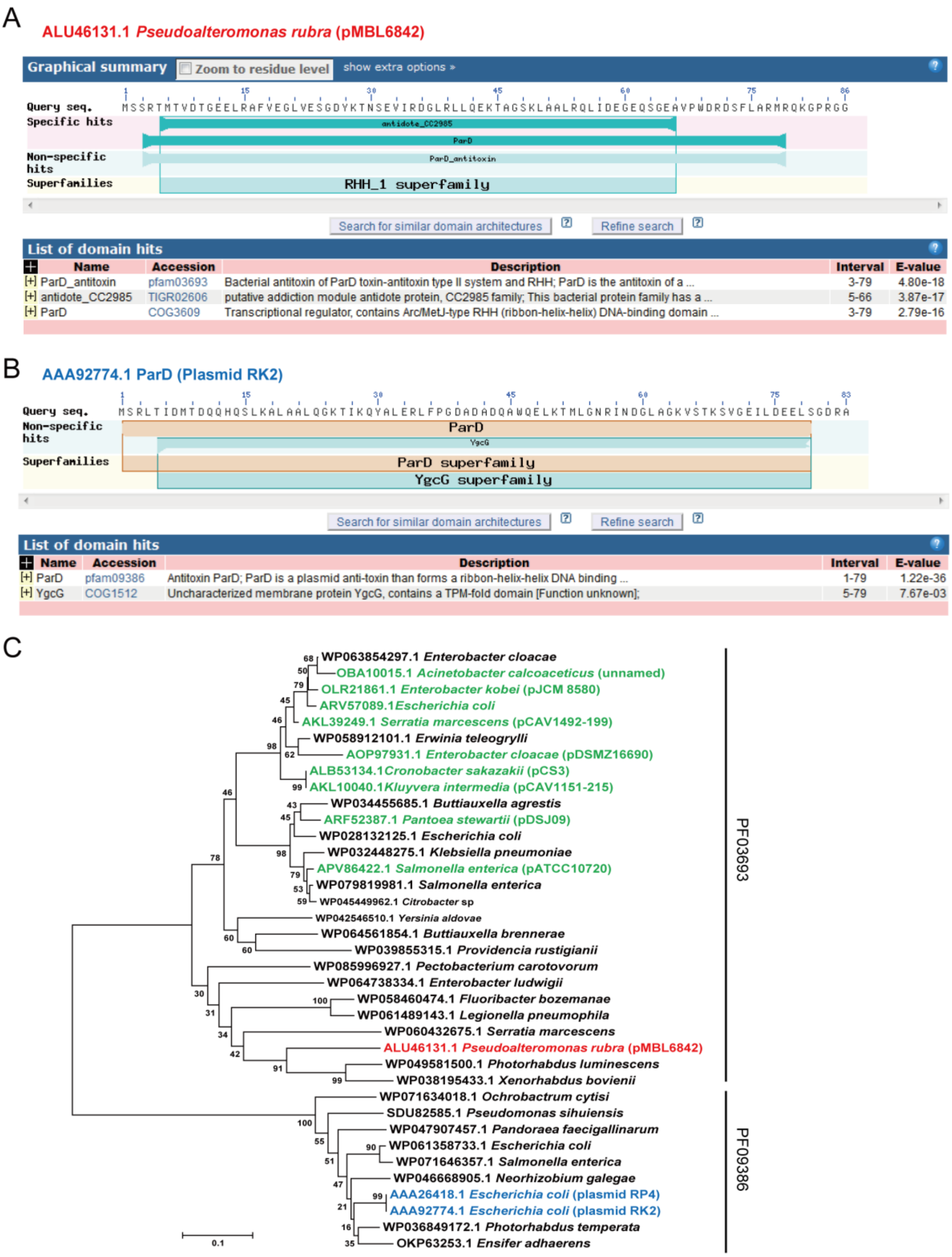

Fig.S1. (A) Sequence analysis of AT705_24525 by BLASTP shows that it belongs in the PF03693 PFAM family. (**B**) ParD of plasmid RK2 belongs in the PF09386 [ParD] family. (**C**) Neighbor-joining phylogenetic tree of 37 ParD antitoxin family proteins based on amino acid (aa) sequences. PF03693 in pMBL6842 is marked in red, other PF03693 homologs (listed in Table 1) from sequenced plasmids are marked in green, and PF09386 in plasmids RK2 and RP4 are marked in blue. Sequences were aligned by MUSCLE program and the dendrogram was constructed by the neighbor-joining method with the MEGA software package (1,000 bootstrap replicates). The numbers indicate the bootstrap coefficients, and the scale bar indicates 0.2 substitutions per aa position.

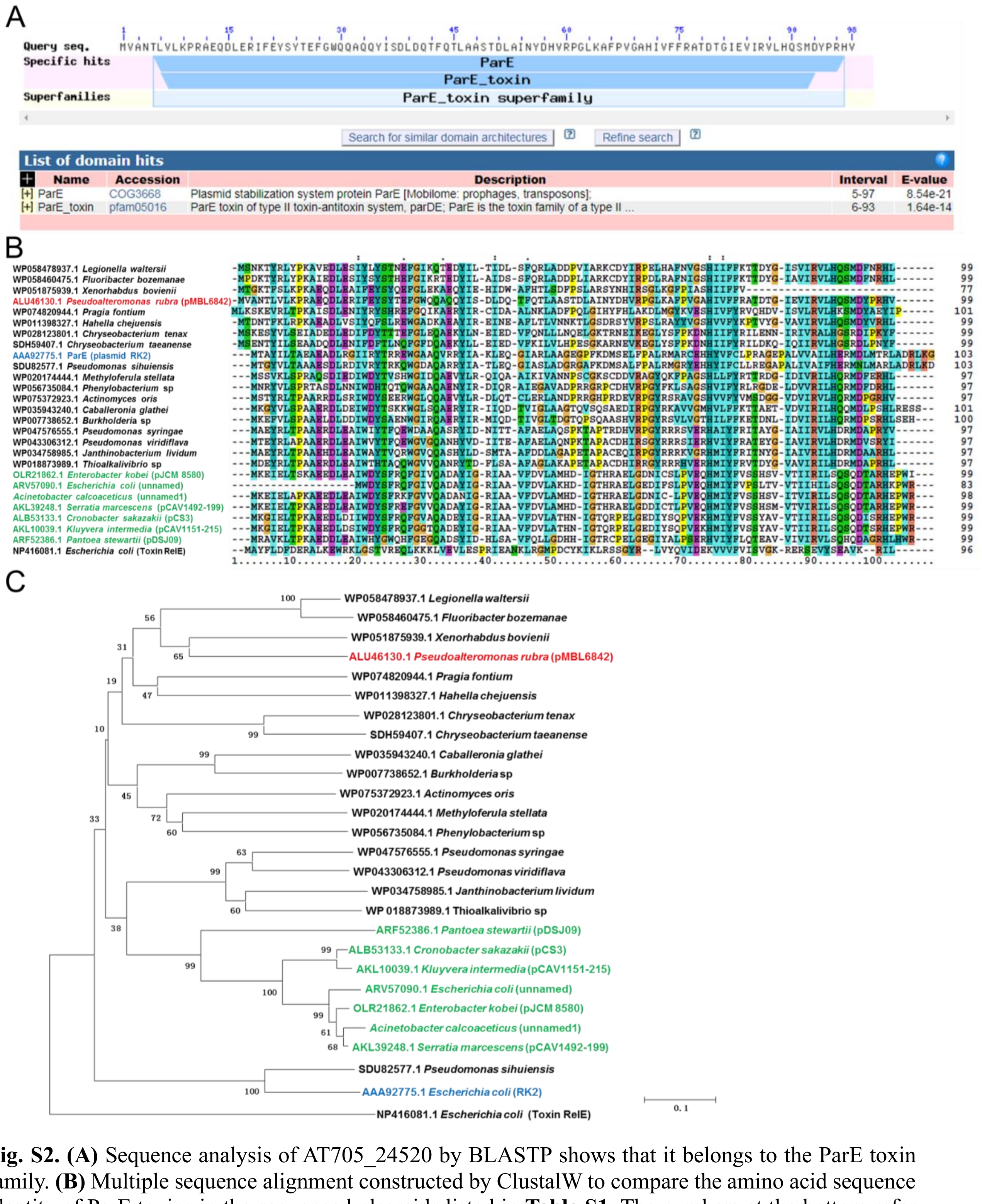

Fig. S2. (A) Sequence analysis of AT705_24520 by BLASTP shows that it belongs to the ParE toxin family. **(B)** Multiple sequence alignment constructed by ClustalW to compare the amino acid sequence identity of ParE toxins in the sequenced plasmids listed in **Table S1**. The numbers at the bottom refer to the amino acid position in the ParE proteins, and identical residues are shaded the same color. **(C)** Phylogenetic tree of ParE toxin proteins in (**B**) based on amino acid (aa) sequences. The RelE toxin in *E. coli* K-12 MG1655 was used as the outgroup. The dendrogram was constructed by the neighbor-joining method with the MEGA software package MUSCLE program (1,000 bootstrap replicates). The numbers indicate the bootstrap coefficients, and the scale bar indicates 0.2 substitutions per aa position.

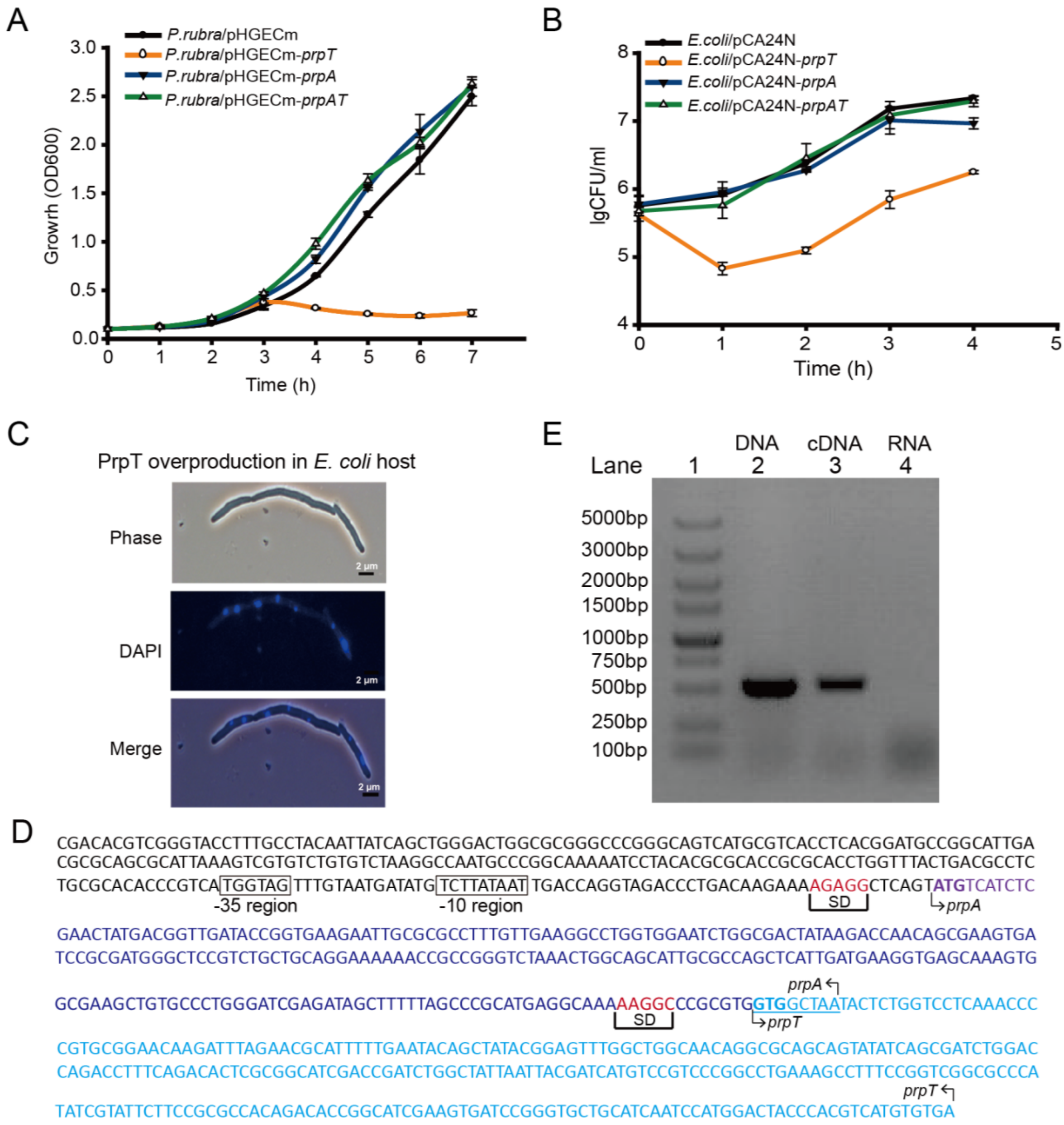

Fig. S3. (**A**) Growth of cells overexpressing *prpA*, *prpT* and *prpA*-*prpT* via pHGECm-based plasmids in *P. rubra*. (**B**) Viability of cells expressing *prpA*, *prpT* and *prpA*-*prpT* via pCA24N-based plasmids in *E. coli*. (**C**) Morphologies of cells overexpressing *prpT* via pCA24N-*prpT* with 0.5 mM IPTG for 2 h were examined by phase contrast microscopy (lower panel) and DAPI staining (middle panel). (**D**) Sequence analysis of *prpA-prpT* operon. The -35 and -10 boxes predicted from BPROM in Softberry were shown in box on the sequences, and the SD (Shine-Dalgano) sequences of *prpA* and *prpT* are highlighted in red. The overlapped coding region of *prpA* and *prpT* is underlined, and the start codon of PrpA and PrpT are marked. (**E**) Co-transcription of *prpA* and *prpT*. Primers were designed to amplify the whole coding region of *prpA* and *prpT*. Approximately 150 ng of cDNA reverse transcribed from *P. rubra* RNA was used as a template, the same amount of *P. rubra* genomic DNA and RNA were used as the positive and negative controls, respectively. Three independent cultures of each strain were tested in **A**-**C**, and the error bars indicate the standard errors of the means (*n* = 3) in **A** and **B**. Only representative images are shown in **C** and **D**.

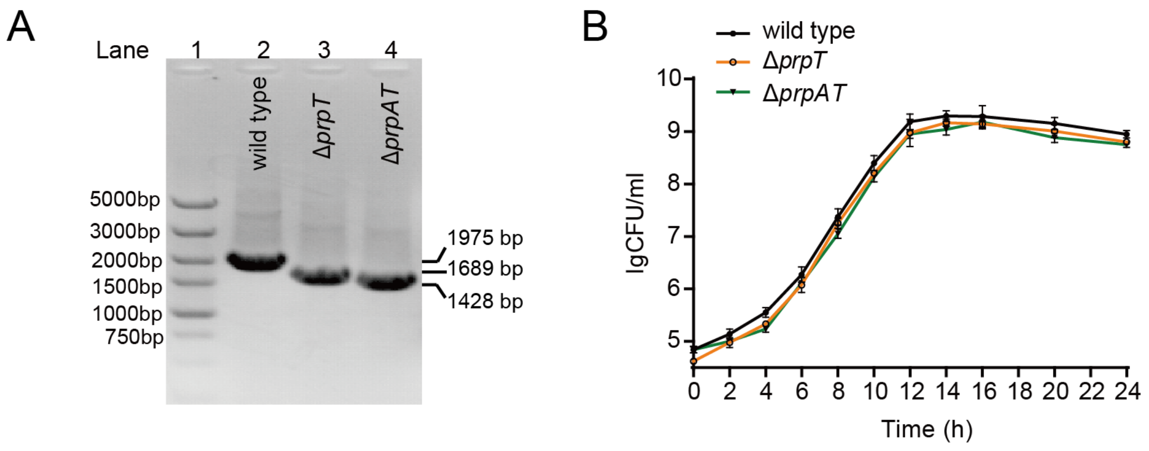

Fig. S4. (**A**) Verification of the *prpT* gene deletion (strain Δ*prpT*) and *prpA*-*prpT* deletion (strain Δ*prpAT*) in *P. rubra* by PCR. **(B)** Colony-forming unit (CFU) of the wild type, Δ*prpT* and Δ*prpAT* strains in 2216E medium (OD_{600}~0.01 at T=0). Cells in exponential (~6 h) and late stationary (~24 h) phases were collected for the determination of plasmid copy number, respectively.

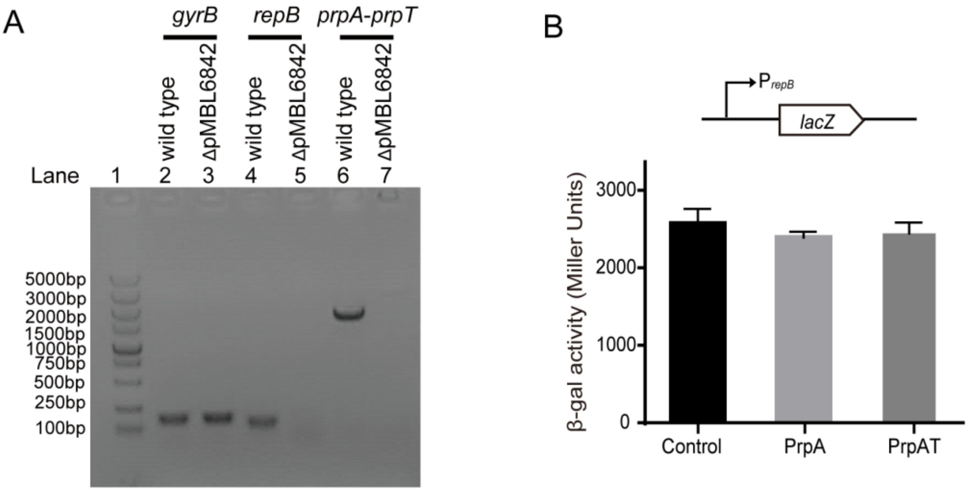

Fig. S5. (**A**) PCR verification of pMBL6842 loss when *repB* is deleted. Chromosomal gene *gyrB*, and plasmid-encoded genes *repB* and *prpA-prpT* were amplified using DNA extracted from the wild type strain and from the mutant strain ΔpMBL6842 as templates with primer pairs qgyrB-F/-R, qrep-F/-R and *prpAT*-con-F/-R. (**B**) The promoter activity of *repB* was measured when overexpressing PrpA (via pCA24N-*prpA*), PrpAT (via pCA24N-*prpAT*), and empty vector (pCA24N). β-galactosidase activity was measured 5 h after adding 0.5 mM IPTG at a turbidity of 0.8 at 600 nm.

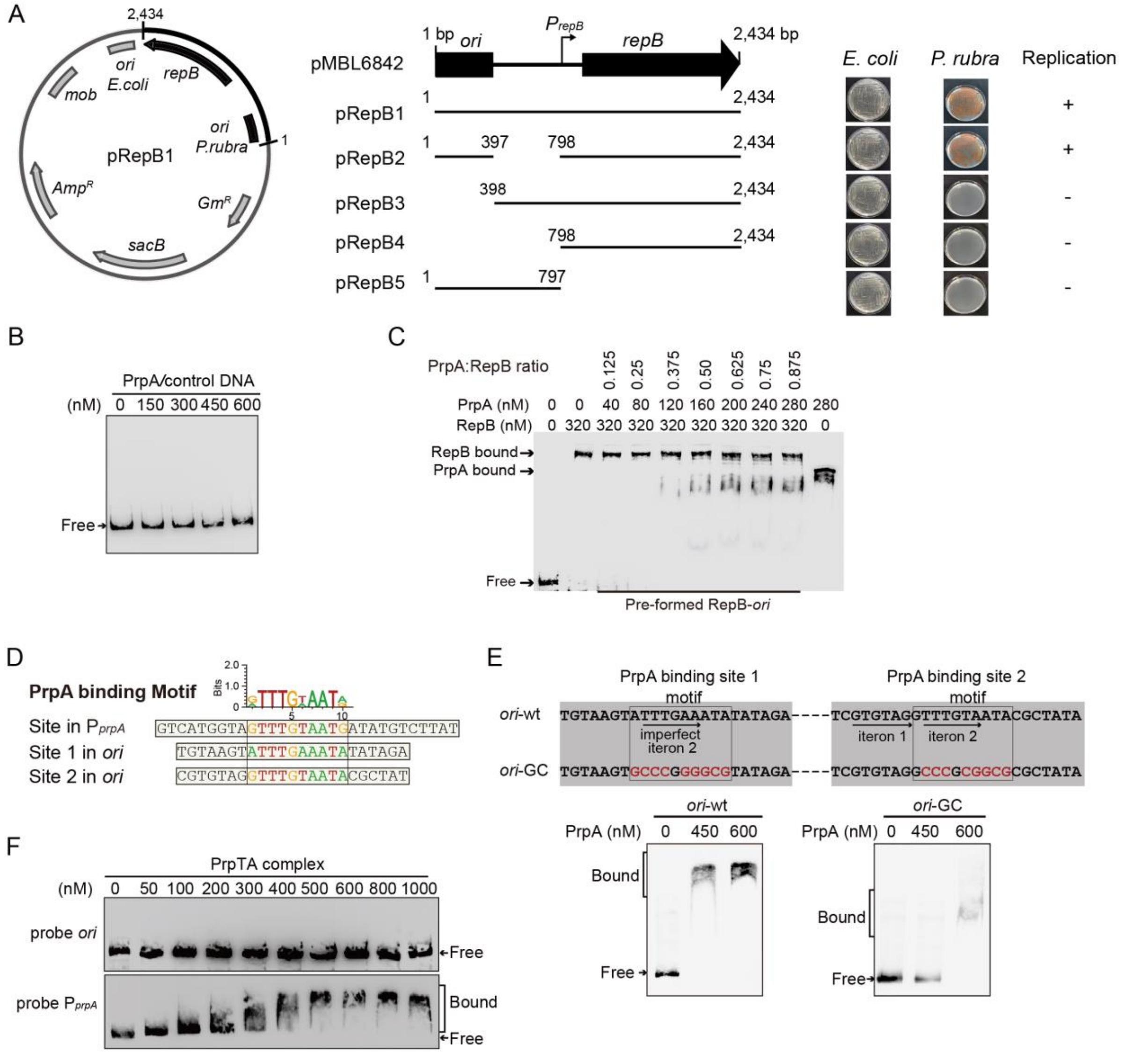

Fig. S6. (**A**) Determination of the pMBL6842 origin (*ori*). Deletion analysis of the pMBL6842 origin (*ori*). Plasmid pRepB1-B5 contained the whole 2.4 kb *ori* fragment or partial *ori* fragments. All the plasmids can replicate in *E. coli* WM3064 host. The "+" or "-" shows the replication ability of each plasmid in *P. rubra* ΔpMBL6842. (**B**) EMSA results show that PrpA did not bind and shift the control DNA. (**C**) EMSA results show that PrpA was unable to dislodge RepB bound to *ori* DNA if less than 1:1 ratio (**D**) Generating the PrpA binding motif by comparing the binding sites of PrpA in the *prpAT* promoter and pMBL6842 *ori*. (**E**) EMSA results show mutating the motif greatly reduces PrpA binding to *ori*. (**F**) EMSA results show that the PrpT/PrpA complex does not bind or shift *ori* compared with the *prpAT* promoter.

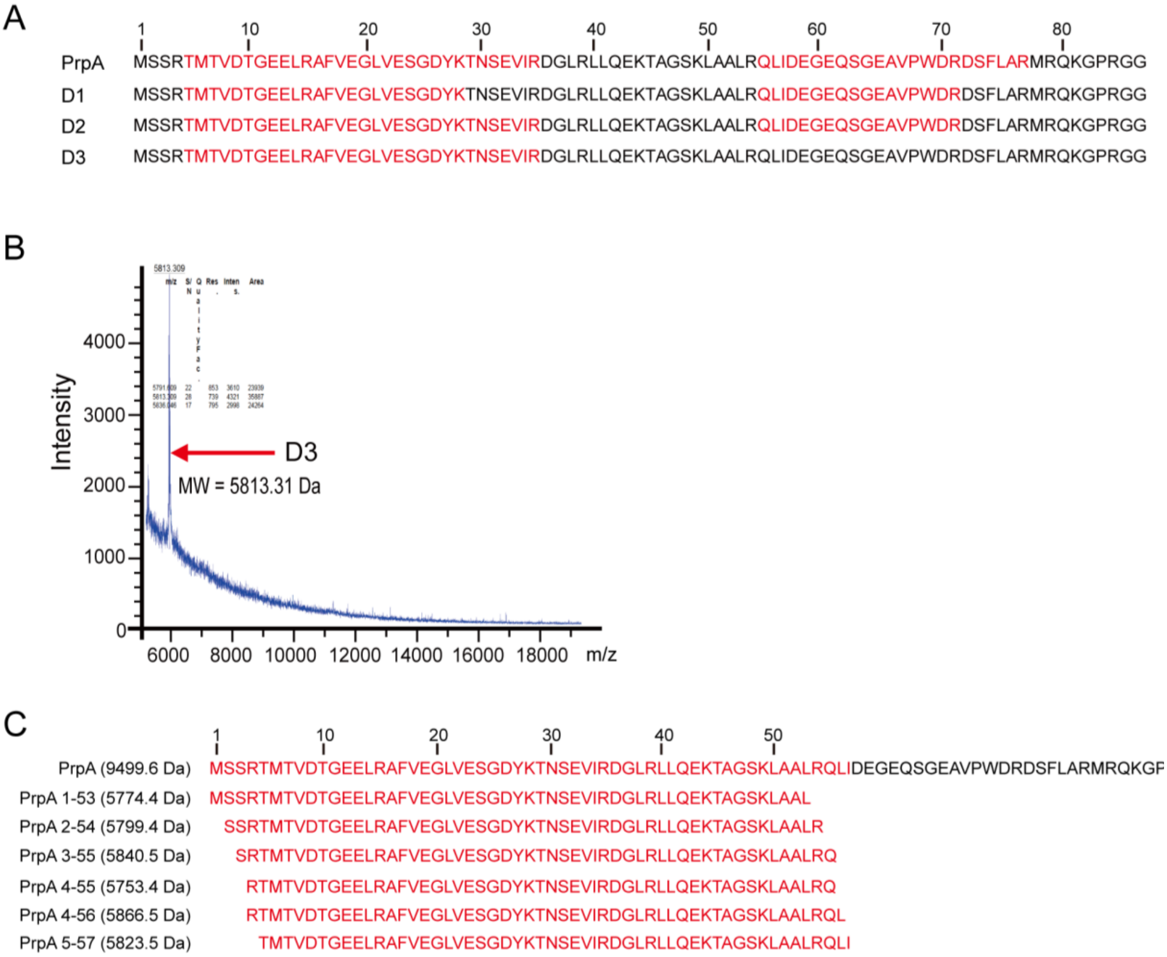

Fig. S7. (**A**) Mass spectrometry analysis of D1, D2 and D3 for partially degraded antitoxin PrpAs as shown in **Fig. 6A**; the full-length PrpA was used as a positive control. (**B**) The molecular weight of degraded PrpA D3 was analyzed using MALDI-TOF MS. (**C**) The end of the C-terminus of D3 was between the 53-57 aa of PrpA (all close to 5.8 kDa for 1-53 aa, 2-54 aa, 3-55 aa, 4-55 aa, 4-56 aa or 5-57 aa).

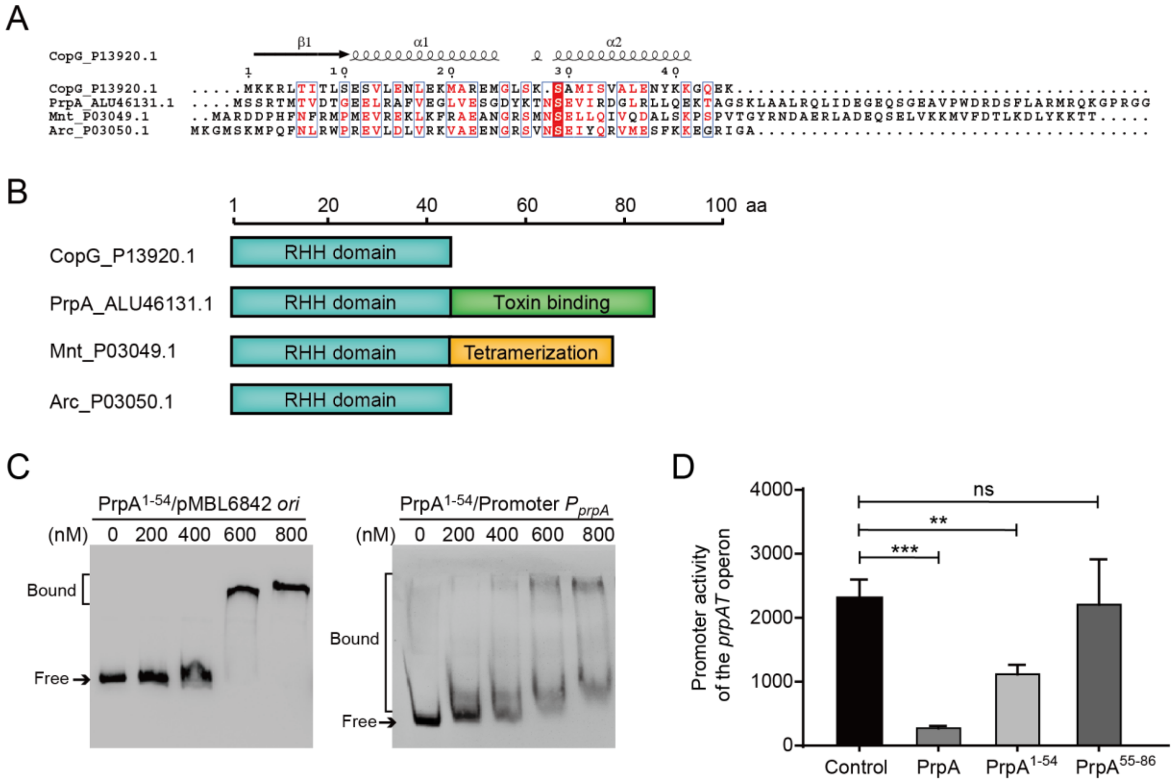

Fig. S8. (**A**) Secondary structure of PrpA and three previously-characterized RHH regulatory proteins. The numbers at the top refer to the aa position in the CopA protein. Conserved residues are boxed, the highly-conserved turn connecting two helices is shaded in black, and non-conserved residues are marked in red. CopG is from the *Streptococcus agalactiae* pMV158 plasmid (P13920.1), Arc is from *Enterobacteria* phage P22 (P03050.1) and Mnt is from *Enterobacteria* phage P22 (P03049.1). (**B**) Schematic of the modular organization of PrpA compared with three previously-characterized RHH regulatory proteins. (**C**) EMSA results showed the C-terminal-truncated PrpA (PrpA$^{1\text{-}54}$) bound and shifted the pMBL6842 *ori* and the *prpA-prpT* promoter. (**D**) The promoter activity of *prpA* was measured when overexpressing full length PrpA, truncated PrpA$^{1\text{-}54}$, PrpA$^{55\text{-}86}$, and empty vector. β-galactosidase activity was measured 6 h after adding 0.5 mM IPTG at OD_{600} of 0.5.

Table S1. ParE/PF03693 (PrpT/PrpA) TA pairs and closely-related TA pairs in large, sequenced plasmids (> 50 kb).

Host strain	Plasmid	GenBank ID	Plasmid size (bp)	PF03693 length /similarity	ParE length /similarity	Conjugation gene cluster	Description
Salmonella enterica	pATCC10720	CP019182.1	124,201	88 aa, 80%	95 aa, 54%	+	Abortive phage infection
Pantoea stewartii	pDSJ09	CP017590.1	132,938	88 aa, 77%	98 aa, 50%	+	-
Enterobacter kobei	pJCM_8580	MKXD01000001.1	122,368	86 aa, 78%	98 aa, 57%	+	Copper resistance
Escherichia coli	Plasmid unnamed	CP019561.1	148,529	86 aa, 78%	82 aa, 53%	+	Copper resistance, TetA
Serratia marcescens	pCAV1492-199	CP011641.1	199,444	86 aa, 78%	98 aa, 57%	+	Heavy metal resistance
Cronobacter sakazakii	pCS3	CP012256.1	53,383	86 aa, 80%	98 aa, 58%	-	Copper resistance
Kluyvera intermedia	pCAV1151-215	CP011600.1	215,092	86 aa, 80%	98 aa, 58%	+	Heavy metal resistance
Enterobacter cloacae	pDSMZ16690	CP017185.1	151,583	86 aa, 76%	98 aa, 76%	+	Carbapenem resistance (14)
Pseudoalteromonas rubra	pMBL6842	CP013613.1	69,868	86 aa, 100%	98 aa, 100%	+	H-NS
Yersinia enterocolitica	pYVa127/90	NC_004564.1	66,591	80 aa, 64%	100 aa, 51%	+	T3SS, virulence (15)
Yersinia enterocolitica	pYVe8081	NC_005017.1	67,720	80 aa, 64%	100 aa, 51%	+	T3SS, virulence (16)
Uncultured bacterium	pTP6	NC_007680.1	54,344	89 aa, 67%	104 aa, 40%	+	Mercury resistance (17)
Pseudomonas sp	pADP-1	NC_004956.1	108,845	93 aa, 67%	104 aa, 40%	+	-
Ensifer meliloti	pSmeSM11b	NC_010865.1	181,251	94 aa, 60%	99 aa, 45%	+	Nitrogen-fixing (18)
Yersinia pestis	pCD1	CP000722.1	70,305	91 aa, 51%	99 aa, 49%	+	T3SS, virulence
Enterobacter aerogenes	R751	NC_001735.4	53,423	89 aa, 67%	104 aa, 40%	+	-
-	pB8	NC_007502.1	57,198	89 aa, 67%	104 aa, 40%	+	Multi-drug resistance (19)

-: not available or not found.

Table S2. Strains and plasmids used in this study.

Bacterial strains/plasmids	Description[a]	Source
***E. coli* strains**		
K-12 BW25113	*lacI*^{q} *rrnB*$_{T14}$ Δ*lacZ*$_{WJ16}$ *hsdR514* Δ*araBAD*$_{AH33}$ Δ*rhaBAD*$_{LD78}$	(20)
BL21(DE3)	F^{-}*ompT hsdSB*(rB^{-} mB^{-}) gal dcm λ(DE3) Ω PtacUV5::T7 polymerase	Novagen
WM3064	*thrB1004 pro thi rpsL hsdS lacZ*Δ*M15* RP4-1360) Δ(*araBAD*)567Δ*dapA*1341::[*erm* pir(wt)]	(21)
DH5α	F^{-}Φ80*lacZ*Δ*M15* Δ(*lacZYA-argF*) U169 *recA1 endA1 hsdR17* (rK^{-},mK^{+}) *phoA supE44* λ^{-} *thi*$^{-1}$ *gyrA96 relA1*	Invitrogen
BTH101	F^{-}, *cya-99*, *araD139*, *galE15*, *galK16*, *rpsL1* (Str^{R}), *hsdR2*, *mcrA1*, *mcrB1*	(22)
***P. rubra* strains**		
SCSIO 6842	SCSIO 6842 wild type, containing conjugative plasmid pMBL6842	(10)
ΔpMBL6842	Plasmid pMBL6842 cured mutant constructed by deleting *repB* gene	This study
**Plasmids**		
pMBL6842	69.9 kb native plasmid in strain *P. rubra* SCSIO 6842	(10)
Δ*prpT*	Deletion *prpT* in plasmid pMBL6842	This study
Δ*prpAT*	Deletion *prpA-prpT* operon in plasmid pMBL6842	This study
pMBL6842::*cm*	Integration a Cm resistance gene into the intergenic region of AT705_RS24695 and AT705_RS24700 of plasmid pMBL6842	This study
pMBL6842::*cm*Δ*prpT*	Deletion *prpT* in plasmid pMBL6842::*cm*	This study
pMBL6842::*cm*Δ*prpAT*	Deletion *prpAT* in plasmid pMBL6842::*cm*	This study
pCA24N	Cm^{R}; *lacI*^{q}, IPTG inducible expression vector in *E. coli*	(23)
pCA24N-*prpT*	Cm^{R}; *lacI*^{q}, P$_{T5\text{-}lac}$::*parE*	This study
pCA24N-*prpA*	Cm^{R}; *lacI*^{q}, P$_{T5\text{-}lac}$::*prpA*	This study
pCA24N-*prpAT*	Cm^{R}; *lacI*^{q}, P$_{T5\text{-}lac}$:: *prpA-prpT*	This study
pCA24N-*prpA*$^{1\text{-}54}$	Cm^{R}; *lacI*^{q}, P$_{T5\text{-}lac}$::*prpA*$^{1\text{-}54}$	This study
pCA24N-*prpA*$^{55\text{-}86}$	Cm^{R}; *lacI*^{q}, P$_{T5\text{-}lac}$::*prpA*$^{55\text{-}86}$	This study
pHGECm	pHGE-P$_{tac}$, Cm^{R}, IPTG inducible expression vector in *P. rubra*	(24)
pHGECm-*prpT*	Cm^{R}; over-expression vector for *prpT*	This study
pHGECm-*prpA*	Cm^{R}; over-expression vector for *prpA*	This study
pHGECm-*prpAT*	Cm^{R}; over-expression vector for *prpA-prpT*	This study

pBBR1Ery	Ery^R, Kan^R; Broad-host-range mobilizable plasmid	(2)
pBBR1Ery-*prpA*	Ery^R, Kan^R; pBBR1Ery containing *prpA* and its own promoter	This study
pBBR1Ery-*prpAT*	Ery^R, Kan^R; pBBR1Ery containing *prpA-prpT* operon and its own promoter	This study
pET28b	Kan^R, *lacI*^{q}, expression vector	Novagen
pET28b-*prpA*-His	Kan^R, *lacI*^{q}, pET28b $P_{T7\text{-}lac}$::*prpA* with C-terminal His-tagged	This study
pET28b-*prpAT*-His	Kan^R, *lacI*^{q}, pET28b $P_{T7\text{-}lac}$::*prpA-prpT* with C-terminal His-tagged for *prpT*	This study
pET28b-N^{FLAG}-*prpA*-C^{His}	Kan^R, *lacI*^{q}, pET28b $P_{T7\text{-}lac}$::*prpA* with C-terminal FLAG-tagged and C-terminal His-tagged	This study
pET28b-*prpA*$^{1\text{-}54}$-His	Kan^R, *lacI*^{q}, pET28b $P_{T7\text{-}lac}$::*prpA*$^{1\text{-}54}$ with C-terminal His-tagged	This study
pET28b-*repB*-His	Kan^R, *lacI*^{q}, pET28b $P_{T7\text{-}lac}$::*repB* with C-terminal His-tagged	This study
pET28b-RBS^{prpA}-N^{FLAG}-PrpAT	Kan^R, *lacI*^{q}, pET28b $P_{T7\text{-}lac}$::*prpAT* with the RBS of PrpA and a N-terminal FLAG-tagged PrpA	This study
pET28b-RBS^{prpA}-PrpAT- C^{FLAG}	Kan^R, *lacI*^{q}, pET28b $P_{T7\text{-}lac}$::*prpAT* with the RBS of PrpA and C-terminal FLAG-tagged PrpT	This study
pHGM01	Gm^R, Amp^R, *sacB*, *ori*-R6K, sucide plasmid for generating in-frame deletions	(3)
pHGM01-*prpT*	Gm^R, pHGM01 containing the homologous arms of *prpA*	This study
pHGM01-*prpAT*	Gm^R, pHGM01 containing the homologous arms of *prpA-prpT* operon	This study
pHGM01-*cm*	Gm^R, Cm^R, pHGM01 containing a Cm resistance gene with the intergenic region between AT705_RS24695 and AT705_RS24700 as the homologous arms	This study
pHGM01-*repB*	Gm^R, pHGM01 containing the homologous arms of *repB*	This study
pMD19-T	Amp^R, *E.coli* cloning vector	Invitrogen
pMD19-T-P_{prpAT}	Amp^R, pMD19-T containing the promoter region of *prpAT*	This study
pMD19-T-P_{repB}	Amp^R, pMD19-T containing the promoter region of *repB*	This study
pMD19-T-*ori*	Amp^R, pMD19-T containing 397 bp *ori* region	This study
pMD19-T-*ori*-GC	Amp^R, pMD19-T containing 397 *ori* where A/T was mutated to G/C in PrpA binding motif	This study
pKT25	Kan^R, encoding T25 fragment	(13)
pUT18C	Amp^R encoding T18 fragment	(13)
pKT25-*prpT*	*prpT* was fused to the C termini of adenylate cyclase in pKT25	This study
pKT25-*prpA*	*prpA* was fused to the C termini of adenylate cyclase in pKT25	This study
pUT18C-*prpA*	*prpA* was fused to the C termini of adenylate cyclase in pUT18C	This study
pUT18C-*prpA*$^{6\text{-}86}$	6-86 aa of *prpA* was fused to the C termini of adenylate cyclase in pUT18C	This study

pUT18C-*prpA*$^{1\text{-}54}$	1-54 aa of *prpA* was fused to the C termini of adenylate cyclase in pUT18C	This study
pUT18C-*prpA*$^{55\text{-}68}$	55-86 aa of *prpA* was fused to the C termini of adenylate cyclase in pUT18C	This study
pHGR01	Kan^R, R6K *ori*, promoterless-*lacZ* reporter vector	(26)
pRBS^{prpA}	Fuse *prpAT* promoter with *lacZ* in pHGR01	This study
pRBS^{prpT}	Replace the RBS^{prpA} with RBS^{prpT} in pRBS^{prpA}	This study
pHGR01-P$_{repB}$	Fuse *repB* promoter with *lacZ* in pHGR01	This study

[a]Gm^R, Cm^R, Kan^R, Ery^R and Amp^R indicate gentamicin, chloramphenicol, kanamycin, erythrocin and ampicillin resistance, respectively.

Table S3. Oligonucleotides used for the construction of plasmids, for mutant strains, and for DNA sequencing. F indicates the forward primer and R indicates the reverse primer. Restriction enzyme sites and vector sequence for one-step recombination are underlined.

Purpose/Primer name	Sequence (5'-3')	Purpose
	**Primers used for TA characterization**	
pHGECm-PrpA-F	CCCGGAATTCATGTCATCTCGAACTATGACGGTT	
pHGECm-PrpA-R	CCGCTCGAGTTAGCCACCACGCGGGCCTTTTTG	pHGECm-*prpA*, pHGECm-*prpT* or pHGECm-*prpAT*
pHGECm-PrpT-F	CCGGAATTCGTGGTGGCTAATACTCTGGTCCTCA	
pHGECm-PrpT-R	CCGCTCGAGTCACACATGACGTGGGTAGTCCATG	
pBBR1Ery-PrpA-F	CCGGAATTCAACGTGCTCATTGTGTTAGGGCTG	pBBR1Ery-*prpA* or pBBR1Ery-*prpAT*
pBBR1Ery-PrpA-R	CCGCTCGAGTTAGTGATGGTGATGGTGGTGGCCACCACGCGGGCCTT	
pBBR1Ery-PrpAT-R	CCGCTCGAGTCAGTGATGGTGATGGTGGTGCACATGACGTGGGTAGT	
pCA24N-PrpA-F	ATACGGATCCGGCCCTGAGGATGTCATCTCGAACTATGACG	
pCA24N-PrpA-R	ACCCTTAGCGGCCGCATAGGTTAGCCACCACGCGGGCCTTTTTGC	pCA24N-*prpA*, pCA24N-*prpT* and pCA24N-*prpAT*
pCA24N-PrpT-F	ATACGGATCCGGCCCTGAGGGTGGTGGCTAATACTCTGGTCCTCA	
pCA24N-PrpT-R	ACCCTTAGCGGCCGCATAGGTCACACATGACGTGGGTAGTC	
	**Primers used for promoter activity assays**	
pRBS^{prpA}-F	CCGGAATTCCCATATTACATATCTCACCACG	
pRBS^{prpA}-R	CCCAAGCTTGTAATCATGGTCATACTGAGCCTCTTTTCTTGTCA	pRBS^{prpA} or pRBS^{prpT}
pRBS^{prpT}-R	CCCAAGCTTGTAATCATGGTCACCACGCGGGCCTTTTTGCCTCATACTGAGCCTCTTTTCTT GTCA	
pHGR01-P$_{repB}$-F	CCGGTAGTCAATAAACCGGTGCACTGAGGTGAAAAATATGCG	pHGR01-P$_{repB}$
pHGR01-P$_{repB}$-R	AGTGCCAAGCTTGTCGACGGATCAATCATGGTCATTTTACTACACAATATCCATTG	
pCA24N-PrpA-F	ATACGGATCCGGCCCTGAGGATGTCATCTCGAACTATGACG	pCA24N-*prpA*$^{1-54}$
pCA24N-PrpA$^{1-54}$-R	ACCCTTAGCGGCCGCATAGGTTAGCGCAATGCTGCCAGTTTAGAC	
pCA24N-PrpA$^{55-86}$-F	ATACGGATCCGGCCCTGAGGATGCAGCTCATTGATGAAGGTGAG	pCA24N-*prpA*$^{55-86}$

pCA24N-PrpA-R	ACCCTTAGCGGCCGCATAGGTTAGCCACCACGCGGGCCTTTTTGC	
**Primers for protein expression and purification**		
pET28b-PrpA-F	TAACTTTAAGAAGGAGATATACATGTCATCTCGAACTATGACGGT	pET28b-*prpA*-His, pET28b-*prpA*$^{1\text{-}54}$-His and pET28b-*prpAT*-His, pET28b-N^{FLAG}-*prpA*-C^{His}
pET28b-PrpA-His-R	GATGATGGCTGCTGCCCATGTTAGTGATGGTGATGGTGGTGGCCACCACGCGGGCCTTT	
pET28b-PrpA$^{1\text{-}54}$-His-R	GATGATGGCTGCTGCCCATGTTAGTGATGGTGATGGTGGTGGCGCAATGCTGCCAGTTT	
pET28b-PrpT-His-R	GATGATGGCTGCTGCCCATGTCAGTGATGGTGATGGTGGTGCACATGACGTGGGTAGTC	
pET28b-FLAG-*prpA*-F	TTAAGAAGGAGATATACATGGATTACAAGGATGACGACGATAAGTCATCTCGAACTATG ACGGT	
pET28b-RepB-F	TTAAGAAGGAGATATACATGATAAGAAAAACTGTGACTC	pET28b-*repB*-His
pET28b-RepB-R	GATGATGGCTGCTGCCCATGTCAGTGATGGTGATGGTGGTGCTTCTTAACCAGCTGTTC	
pET28b-RBS^{prpA}-N^{FLAG}-PrpAT-F1	ATGGATTACAAGGATGACGACGATAAGTCATCTCGAACTATGACGGTTG	pET28b-RBS^{prpA}-N^{FLAG}-PrpAT
pET28b-RBS^{prpA}-N^{FLAG}-PrpAT-F2	TGACAAGAAAAGAGGCTCAGTATGGATTACAAGGATGACGAC	
pET28b-RBS^{prpA}-N^{FLAG}-PrpAT-F3	GTGAGCGGATAACAATTCCCCTTGACAAGAAAAGAGGCTCAGT	
pET28b-RBS^{prpA}-N^{FLAG}-PrpAT-R	GATGATGATGGCTGCTGCCCATGTCACACATGACGTGGGTAGTCCATG	
pET28b-RBS^{prpA}-PrpAT-C^{FLAG}-F1	TGACAAGAAAAGAGGCTCAGTATGTCATCTCGAACTATGACG	pET28b-RBS^{prpA}-PrpAT-C^{FLAG}
pET28b-RBS^{prpA}-PrpAT-C^{FLAG}-F2	GTGAGCGGATAACAATTCCCCTTGACAAGAAAAGAGGCTCAGT	
pET28b-RBS^{prpA}-PrpAT-C^{FLAG}-R1	TCACTTATCGTCGTCATCCTTGTAATCCACATGACGTGGGTAGTCCATG	
pET28b-RBS^{prpA}-PrpAT-	GATGATGATGGCTGCTGCCCATGTCACTTATCGTCGTCATCCTTG	

C^{FLAG}-R2		
**Primers for bacterial two-hybrid (BACTH) assays**		
pKT25-PrpT-F	CGGGGTACCTGCTAATACTCTGGTCCTCAAACCCC	pKT25-*prpT*
pKT25-PrpT-R	CGGGGTACCTCACACATGACGTGGGTAGTCCATG	
pKT25-PrpA-F	CGGGGTACCTTCATCTCGAACTATGACGGTTGAT	pKT25-*prpA*
pKT25-PrpA-R	CGGGGTACCTTAGCCACCACGCGGGCCTTTTTG	
pUT18C-PrpA-F	CGGGGTACCGTCATCTCGAACTATGACGGTTGAT	pUT18C-*prpA*, pUT18C-*prpA*$^{6\text{-}86}$, pUT18C-*prpA*$^{1\text{-}54}$ or pUT18C-*prpA*$^{55\text{-}86}$
pUT18C-PrpA-R	CCGGAATTCTTAGCCACCACGCGGGCCTTTTTG	
pUT18C-PrpA$^{6\text{-}86}$-F	CGGGGTACCGACGGTTGATACCGGTGAAGAATTGC	
pUT18C-PrpA$^{1\text{-}54}$-R	CCGGAATTCTTAGCGCAATGCTGCCAGTTTAG	
pUT18C-PrpA$^{55\text{-}86}$-F	CGGGTACCGCGCCAGCTCATTGATGAAGGT	
**Primers for identification the replication origin of pMBL6842**		
pRepB1-F	GGGGACAAGTTTGTACAAAAAAGCAGGCTAAAATAGGGGGTCGTACTTACTTTG	pRepB1, pRepB2, pRepB3, pRepB4 or pRepB5
pRepB1-R	GGGACCACTTTGTACAAGAAAGCTGGGTTCACTTCTTAACCAGCTGTTCTTC	
pRepB2-mid-R	CACAAATACAGAGGGACAACACCTAAGGTGTAGTGTTGTACGAAT	
pRepB2-mid-F	ATTCGTACAACACTACACCTTAGGTGTTGTCCCTCTGTATTTGTG	
pRepB3-F	GGGGACAAGTTTGTACAAAAAAGCAGGCTTGGGGTATTTCCTACACCCAGAG	
pRepB4-F	GGGGACAAGTTTGTACAAAAAAGCAGGCTGTGTTGTCCCTCTGTATTTGTG	
pRepB5-R	GGGACCACTTTGTACAAGAAAGCTGGGTGTGTTCAGGATCAGCACGCTCC	
**Primers for probe amplification of EMSA and DNase I footprinting assays**		
ori-probe-F	AAAATAGGGGGTCGTACTTAC	Used in Figure 5C-G or Fig 6C
ori-probe-R	CTAAGGTGTAGTGTTGTACGA	
P_{prpA}-probe-F	CTTTTCAACCCGTTGTTCATTA	Used in Figure 3D-F or Fig 6C
P_{prpA}-probe-R	ACTGAGCCTCTTTTCTTGTCAG	

oriGC-up-R1	TATACGCCCCGGGCACTTACAATTTATAGCACTAGATCGATCTG	Mutate A/T to G/C in PrpA binding sites
oriGC-up-R2	TACACCATAGAATTATTATATCTATACGCCCCGGGCACTTA	
oriGC-up-R3	CCTACACGATGCCGTACAAAGCTACACCATAGAATTATTATATC	
oriGC-up-F1	CCCGCGGCGCGCTATAGTGTAGCTTTGTACGGTTTGC	
oriGC-up-F2	GCTTTGTACGGCATCGTGTAGGCCCGCGGCGCGCTATAG	
**Primers for the construction of deletion mutants in SCSIO 6842**		
prpAT-up-F	GGGGACAAGTTTGTACAAAAAAGCAGGCTCACAAGGCACCCTGATACTGGAC	pHGM01-*prpA* or pHGM01-*prpAT*
prpAT-up-R	GGTCCGGGTTCGCTATCTATACTGAGCCTCTTTTCTTGTCAG	
prpAT-down-F	ATAGATAGCGAACCCGGACCCGTCCCCCGACTGCGGGCCCTA	
prpAT-down-R	GGGACCACTTTGTACAAGAAAGCTGGGTGATCTCCTCGTTGCCTCTGTCC	
prpT-down-F	GGTCCGGGTTCGCTATCTATTTAGCCACCACGCGGGCCTTTTTG	
Cm-up-F	GGGGACAAGTTTGTACAAAAAAGCAGGCTCAGTTTCACAGTTATGTGTTG	pHGM01-*cm*
Cm-up-R	GTGATCTTCCGTCACAGGTAGTCAATTCATTCAACCGTTG	
Cm-F	CAACGGTTGAATGAATTGACTACCTGTGACGGAAGATCAC	
Cm-R	TTAATTAAATCAAACGCTTATTACGCCCCGCCCTGCCACT	
Cm-down-F	AGTGGCAGGGCGGGGCGTAATAAGCGTTTGATTTAATTAA	
Cm-down-R	GGGACCACTTTGTACAAGAAAGCTGGGTGAGACACCTTTAATATCCTG	
RepB-up-F	GCTCTAGATGGCATCGAGGTGGACTT	pHGM01-*repB*
RepB-up-R	CCGGAATTCGCAACGGGTTACGGAGGT	
RepB-down-F	CCGGAATTCACAAATACAGAGGGACAAC	
RepB-down-R	CCCAAGCTTTTTCATATCCGTAAGCAG	
**Primers for verification of the constructed plasmids and deletion mutant strains**		
pHGECm-F	CACCTCGCTAACGGATTCACC	
pHGECm-R	ACACTACCATCGGCGCTACG	
pCA24N-F	GATAACAATTTCACACAGAATT	

pCA24N-R	GTCAGAGGTTTTCACCGTCATCA
pHGR01-F	CGTCAATTATTACCTCCACG
pHGR01-R	GTGCTGCAAGGCGATTAAG
pET28b-F	TAATACGACTCACTATAGGG
pET28b-R	TATGCTAGTTATTGCTCAG
pKT25-F	CGCATCTGTCCAACTTCCGC
pKT25-R	CGCCAGGGTTTTCCCAGTCA
M13F	CGCCAGGGTTTTCCCAGTCACGAC
M13R	AGCGGATAACAATTTCACACAGGA
pUT18C-F	GCGAGGGCTATGTCTTCTACG
pUT18C-R	GGGCTGGCTTAACTATGCGG
prpAT-con-F	CTGCAAGCCTGTTCAACGCCAC
prpAT-con-R	GCCATATTCTCTGTGCTATTGG
Cm-con-F	CTATGGATAAACCGTTCGAC
Cm-con-R	GTCCGAGGTGATGCGATTTG
**Primers used for qPCR**	
qgyrB-F	CAAGCAACGCAACAGGTGAT
qgyrB-R	CACCCGGGTTTTCCAGTAGA
qrep-F	CTGTCAACCCAGCACCATGT
qrep-R	AATGGCTTTTTGCGGTTGT

S1 References

1. I. A. Chen, *et al.*, IMG/M v.5.0: an integrated data management and comparative analysis system for microbial genomes and microbiomes. *Nucleic Acids Res* **47**, D666-D677 (2019).
2. P. Wang, *et al.*, Development of an efficient conjugation-based genetic manipulation system for *Pseudoalteromonas*. *Microb Cell Fact* **14**, 11 (2015).
3. M. Jin., *et al.*, Unique organizational and functional features of the cytochrome c maturation system in *Shewanella oneidensis*. *PLoS One* **8**, e75610 (2013).
4. J. H. Miller, Experiments in molecular genetics. *Cold Spring Harbor, NY: Cold Spring Harbor Laboratory Press* (1972).
5. F. H. Stephenson, Cell growth. Calculations for molecular miology and biotechnology.43–79 (2016).
6. H. Wang, *et al.*, Increased plasmid copy number is essential for *Yersinia* T3SS function and virulence. *Science* **353**, 492-495 (2016).
7. X. Liu, *et al.*, Physiological function of Rac prophage during biofilm formation and regulation of Rac excision in *Escherichia coli* K-12. *Sci Rep* **5**, 16074 (2015).
8. H. Schagger, Tricine-SDS-PAGE. *Nature Protocols* **1**, 16-22 (2006).
9. D. N. Perkins, D. J. C. Pappin, D. M. Creasy, J. S. Cottrell, Probability-based protein identification by searching sequence databases using mass spectrometry data. *Electrophoresis* **20**, 3551-3567 (1999).
10. B. Li, *et al.*, Complete genome sequence of *Pseudoalteromonas rubra* SCSIO 6842, harboring a putative conjugative plasmid pMBL6842. *J Biotech* **224**, 66-67 (2016).
11. Y. Kim, *et al.*, *Escherichia coli* toxin/antitoxin pair MqsR/MqsA regulate toxin CspD. *Environ Microbiol* **12**, 1105-1121 (2010).
12. Y. Guo, *et al.*, Antitoxin HigA inhibits virulence gene *mvfR* expression in *Pseudomonas aeruginosa*. *Environ Microbiol* **21**, 2707-2723 (2019).
13. G. Karimova, J. Pidoux, A. Ullmann, D. Ladant, A bacterial two-hybrid system based on a reconstituted signal transduction pathway. *Proc Natl Acad Sci U S A* **95**, 5752-5756 (1998).
14. K. D. Chavda, *et al.*, Comprehensive genome analysis of carbapenemase-producing *Enterobacter spp.*: new insights into phylogeny, population structure, and resistance mechanisms. *mBio* **7**, (6) (2016).
15. N. J. Snellings, M. Popek, L. E. Lindler, Complete DNA sequence of *Yersinia enterocolitica* serotype 0:8 low-calcium-response plasmid reveals a new virulence plasmid-associated replicon. *Infect Immun* **69**, 4627-4638 (2001).
16. D. A. Portnoy, H. Wolf-Watz, I. Bolin, A. B. Beeder, S. Falkow, Characterization of common virulence plasmids in *Yersinia* species and their role in the expression of outer membrane proteins. *Infect Immun* **43**, 108-114 (1984).
17. K. Smalla, *et al.*, Increased abundance of IncP-1beta plasmids and mercury resistance genes in mercury-polluted river sediments: first discovery of IncP-1beta plasmids with a complex mer transposon as the sole accessory element. *Appl Environ Microbiol* **72**, 7253-7259 (2006).
18. M. Stiens, S. Schneiker, A. Puhler, A. Schluter, Sequence analysis of the 181-kb accessory plasmid pSmeSM11b, isolated from a dominant *Sinorhizobium meliloti* strain identified during a long-term field release experiment. *FEMS Microbiol Lett* **271**, 297-309 (2007).
19. A. Schluter, *et al.*, Plasmid pB8 is closely related to the prototype IncP-1 beta plasmid R751 but transfers poorly to *Escherichia coli* and carries a new transposon encoding a small multidrug resistance efflux protein. *Plasmid* **54**, 135-148 (2005).
20. T. Baba, *et al.*, Construction of *Escherichia coli* K-12 in-frame, single-gene knockout mutants: the Keio collection. *Mol Syst Biol* **2**, 0008 (2006).
21. C. Dehio, M. Meyer, Maintenance of broad-host-range incompatibility group P and group Q plasmids and transposition of Tn5 in *Bartonella henselae* following conjugal plasmid transfer from *Escherichia coli*. *J Bacteriol* **179**, 538-540 (1997).
22. D. Ladant, A. Ullmann, *Bordetella pertussis* adenylate cyclase: a toxin with multiple talents. *Trends Microbiol* **7**, 172-176 (1999).
23. M. Kitagawa., *et al.*, Complete set of ORF clones of *Escherichia coli* ASKA library (A complete

Set of *E. coli* K-12 ORF Archive): unique resources for biological research. *DNA Res* **12**, 291-299 (2005).

24. P. Wang., *et al.*, Dissemination and loss of a biofilm-related genomic island in marine *Pseudoalteromonas* mediated by integrative and conjugative elements. *Environ Microbiol* **19**, 4620-4637 (2017).
25. H. Fu, M. Jin, L. Ju, Y. Mao, H. Gao, Evidence for function overlapping of CymA and the cytochrome bc1 complex in the *Shewanella oneidensis* nitrate and nitrite respiration. *Environ Microbiol* **16**, 3181-3195 (2014).
